# *Lactiplantibacillus plantarum* dfa1 reduces obesity caused by a high carbohydrate diet by modulating inflammation and gut microbiota

**DOI:** 10.1038/s41598-025-10435-x

**Published:** 2025-07-10

**Authors:** Thunnicha Ondee, Krit Pongpirul, Lampet Wongsaroj, Sayamon Senaprom, Suphot Wattanaphansak, Asada Leelahavanichkul

**Affiliations:** 1 Department of Preventive and Social Medicine, Faculty of Medicine, Chulalongkorn University, Bangkok, 10330 Thailand; 2 Center of Excellence in Preventive and Integrative Medicine (CE-PIM), Faculty of Medicine, Chulalongkorn University, Bangkok, 10330 Thailand; 3 Department of International Health, Johns Hopkins Bloomberg School of Public Health, Baltimore, MD 21205 USA; 4 Bumrungrad International Hospital, Bangkok, 10110 Thailand; 5 Department of Infection Biology & Microbiomes, Faculty of Health and Life Sciences, University of Liverpool, Liverpool, L69 3GB UK; 6 Porcinotec Co.,Ltd., Tiwanont Road, Tambon Talat Khwan, Amphur Mueang, Nonthaburi, Thailand; 7 Department of Veterinary Medicine, Faculty of Veterinary Science, Chulalongkorn University, Pathum Wan, Bangkok, Thailand; 8 Department of Microbiology, Faculty of Medicine, Chulalongkorn University, Bangkok, 10330 Thailand; 9 Department of Microbiology, Center of Excellence in Translational Research in Inflammation and Immunology (CETRII), Chulalongkorn University, Bangkok, 10330 Thailand; 10 Nephrology Unit, Department of Medicine, Faculty of Medicine, Chulalongkorn University, Bangkok, 10330 Thailand

**Keywords:** *Lactiplantibacillus plantarum*, Glucose, Sugar, Prediabetes, Obesity, Microbiome, Dysbiosis, Nutrition, Experimental models of disease

## Abstract

Given the potential of probiotics to counteract obesity induced by high glucose diets (HGD) and the beneficial effects of high-fiber diets, this study explored the impact of *Lactiplantibacillus plantarum* dfa1 (Lp dfa1) in mice consuming modified diets, including a unique high-carbohydrate biscuit diet (HBD) featuring Prachuap Khiri Khan’s Pineapple Cheese Cake Biscuit for its rich fiber content. Notably, the fruit-derived soluble fiber HBD might have a synbiotic effect (the enhanced beneficial bacteria in the gut) different from HGD. in The standard mouse diet (RD) with a carbohydrate: protein: fat (C: P:F) ratio of 56:13:31 served as the base, which was then modified with either glucose to form the HGD (C: P:F at 60:25:15) or blended with the high-fiber dessert to create the HBD (C: P:F at 70:9:21). Over 12 weeks, mice were fed HGD or HBD with and without daily oral administration of Lp dfa1 at 1 × 10^9^ CFU. This study aimed to assess the effects on obesity indicators (weight gain, lipid profiles, fat deposition), prediabetes markers (fasting glucose, insulin, oral glucose tolerance test, and Homeostatic Model Assessment for Insulin Resistance (HOMA-IR)), intestinal health (FITC-dextran assay for enterocyte injury, serum cytokines TNF-α, IL-10, and IL-6), and liver health (enzymes, weight, histology, carbohydrate and fat components, and oxidative stress). Both HGD and HBD induced similar obesity metrics, prediabetic conditions, enterocyte injury, altered serum cytokines, and liver damage. Remarkably, Lp dfa1 administration mitigated these adverse effects with an elevation in fecal short-chain fatty acids. Microbiome analysis revealed diet-induced dysbiosis, with a notable difference between HGD and HBD impacts, particularly in the Fermicutes/Bacteroides ratio and *Akkermansia* spp. abundance, which was significantly elevated in the HBD + Lp group. Actinomycota with a lower Proteobacteria in HBD compared with HGD group indicate the possible different impacts between the mixed sugar with fruit-derived soluble fiber versus pure glucose. Interestingly, Lp dfa1 elevated *Akkermansia* spp. (a well-known beneficial probiotic against obesity) only in mice with HBD but not HGD, despite an obesity attenuation by Lp dfa1 in both HGD and HBD groups, implying the different anti-obesity mechanisms. In vitro experiments revealed Lp dfa1’s supernatant reduced inflammation in Caco-2 and HepG2 cell lines, evidenced by improved transepithelial electrical resistance (TEER), expression of occludin-1 (a tight junction molecule), decreased inflammatory mediators (NF-κB, TNF-α, IL-8, IL-6, and IL-10), and elevated mucin gene. Conclusively, Lp dfa1 demonstrates a dual mechanism in preventing sugar-induced intestinal injury and supporting metabolic health: direct cellular protection and microbiome modulation. These findings highlight the additive effects of Lp dfa1 and high-fiber diets, particularly emphasizing the Pineapple Cheese Cake Biscuit-based diet’s role in enhancing gut microbiota and addressing diet-induced health issues.

**Supplementary Information:**

The online version contains supplementary material available at 10.1038/s41598-025-10435-x.

## Introduction

Obesity-induced diabetes mellitus stands as a formidable global health issue, intimately associated with additional complications such as cardiovascular and dyslipidemia disease, which pose dire consequences, especially for patients in a critical state of health^1^. The consumption of high-carbohydrate diets contributes to the prevalence of metabolic syndrome, a condition marked by a constellation of risk factors—impaired fasting blood glucose (FBG), reduced high-density lipoprotein (HDL) levels, elevated triglycerides, hypertension, and obesity^2^ and affects 20–25% of the global population. The syndrome often progresses, to pre-diabetes condition (characterized by high FBG but not reaching diabetes diagnostic levels) or overt type 2 diabetes mellitus, underscoring the risks associated with unhealthy dietary patterns^3^.

The pathophysiology of obesity-induced inflammation is multifaceted, involving adipocyte hypertrophy and apoptosis due to hypoxia^4,5^, alterations in adipokines such as adiponectin reduction and leptin increase^6^mitochondrial damage^7^ and metabolic endotoxemia resulting from gut barrier dysfunction^8^. These mechanisms contribute to serious vascular complications of obesity, including atherosclerosis^9^. As such, the translocation of pathogen molecules from the gut into the blood circulation due to the intestinal permeability defect, referred to as “leaky gut or gut leakage”^10^are mentioned in several conditions, including obesity and diabetes^11–14^. The immune responses against pathogen-associated molecular patterns (PAMPs) that are translocated from the intestines induce the severe response towards the host partly due to the foreignness of the molecules compared to the host molecules^15^.

Dietary composition, particularly high carbohydrate intake, plays a crucial role in shaping gut microbiota dysbiosis^16^—an alteration of organisms in the intestine^17^—is developed partly though the different carbohydrate metabolisms among different groups of bacteria^18,19^, resulting in the selective growth of some bacteria over other microbial groups. This dysbiosis exacerbates gut mucosal damage in obesity and diabetes, evident from increased levels of lipopolysaccharide (LPS; a major component of Gram-negative bacteria), referred to as metabolic endotoxemia^20,21^, that can lead to steatohepatitis and systemic inflammation.

Glucose is widely recognized for its detrimental effects, whereas fructose syrup and sucrose are commonly employed in a multitude of beverages, soft drinks, and commercial ready to eat dessert^22^. Although there are apparent differences between glucose and fructose concerning hepatic lipogenesis and insulin signaling^23^, we recently demonstrate a similar adverse impact of both carbohydrate in obesity and pre-diabetes condition in mice^24^. Additionally, the consumption of both glucose and fructose directly induce intestinal inflammation that leads to gut barrier defect and metabolic endotoxemia with systemic inflammation^24,25^.

Interestingly, the inclusion of non-carbohydrate components, such as fruit-derived soluble fiber, in commercial desserts could modulate the impact of high carbohydrate diets. Specifically, fruit-derived soluble fiber may foster the growth of microbiota, such as the bacteria in phylum Firmicutes (Bacillota) and Bacteroidetes, known for their fiber-fermenting capabilities and subsequent production of beneficial metabolites for the enterocytes in several members of the phylum^26^. However, the additional carbohydrates in these fruits could potentially aggravate obesity. Pineapple, a common dessert ingredient in Thailand, presents an intriguing case for study due to its fiber content.

Due to the attenuation of leaky gut from several causes^20^ by host-beneficial probiotics^27–29^, partly through the anti-inflammation to intestinal integrity and *Lactiplantibacillus plantarum* (previously known as *Lactobacillus plantarum*) are lactic acid-producing bacteria that are frequently used^30^. As such, *L. plantarum* has several beneficial properties, including (i) the well-tolerance against both acid and bile^31^, (ii) the well-known synergy between *L. plantarum* with other probiotics^32^, (iii) the relatively easy preparatory procedure, and (iv) very less adverse effect (reported only in the immune-compromised hosts^33^). Although there are several non-pharmacologic strategies to attenuate obesity (lifestyle modifications, exercise, dietary fiber, and probiotics)^34^, *L. plantarum* dfa1 (Lp dfa1) is the probiotics the probiotics that are isolated from the Thai population^35^ that needs to explore the use in several health conditions. As such, Lp dfa1 previously demonstrates several beneficial effects in high fat diet-induced obesity mice^24^ which might be also helpful in carbohydrate-induced obesity. This study hypothesizes that the adverse effects of a high glucose diet (HGD) and high-carbohydrate biscuit diet (HBD; biscuit cheesecake with pineapple fiber) might be improved by Lp dfa1 partly through anti-inflammatory property and microbiome alteration. Notably, there might be a synbiotic effect from the fermentable fibers in HBD that might enhance the benefits of probiotics. Hence, we tested our hypothesis both in vivo and in vitro experiments.

## Materials and methods

### Animals and procedures

In accordance with US National Institutes of Health guidelines, the animal care and use procedure (SST 025/2563) at Chulalongkorn University Faculty of Medicine in Bangkok, Thailand, was authorized. Male C57BL/6 mice, aged 8 weeks, were procured from Nomura Siam (Pathumwan, Bangkok, Thailand). The standard laboratory chow (Mouse Feed Food No.082, C.P. Company, Bangkok, Thailand) served as the basis for the normal diet (RD), composed of 55.5% carbohydrate (sugar-free), 31.3% protein, and 13.2% fat, with an energy content of 3.04 kcal/g. The high glucose diet (HGD) was a modified version of the usual mouse chow (23.5% protein and 10.0% fat), containing 66.5% carbohydrates and 24.8% glucose, equating to the same energy content of 3.04 kcal/g as the regular diet. For high-carbohydrate biscuit diet (HBD; biscuit cheesecake enriched with pineapple fiber), the high carbohydrate diet was formulated as a modified version of the standard mouse chow (9% protein and 21% fat)^36,37^. Additional information has been provided in the supplementary table description section. In this modified high carbohydrate diet, the energy content was adjusted to match that of the regular diet, calculated at 3.04 kcal/g. This ensured that the energy intake from the high carbohydrate diet was equivalent to that of the standard diet, providing a fair basis for comparison between the two dietary regimens. To determine intake of mice, mouse food was weighed every 3 days and the intake was calculated by the reduction of food weight divided by number of mice in the box.

The probiotic strain Lp dfa1 was isolated from fecal samples obtained from Thai volunteers at Chulalongkorn University’s Faculty of Medicine. This isolation was made possible through funding provided by the Thailand Science Research and Innovation (TSRI: RDG6150124)^25^. Prior to use, the bacterial stock culture was grown on deMan Rogosa Sharpe (MRS) agar under anaerobic conditions using gas generation sachets (Anaero Pack-Anaero, Mitsubishi Gas Chemical, Japan) at 37 °C for 48 h. Subsequently, it was preserved in MRS broth (Oxoid, Hampshire, UK) supplemented with 20% (vol/vol) glycerol and stored at -80 °C. Then, the spectrophotometer (Bio-Rad, Smart Spec 3000; Bio-Rad, Hercules, CA, USA) was utilized at an optical density of 600 nm wavelength (OD600) of 0.15 in 0.5 mL phosphate buffer solution (PBS) or PBS alone, compatible with approximately 1 × 10^9 CFU of bacteria, and orally administered once daily for 12 weeks before being sacrificed with a cardiac puncture under isoflurane anesthesia. The fasting blood of mice (12-hour fasting with free access to drinking water) was tested for glucose (FBG), fructose, insulin, and lipid profile (cholesterol and triglyceride) using several procedures, including the Homeostatic Model Assessment for Insulin Resistance (HOMA-IR or HOMA index), oral glucose tolerance test (OGTT), insulin tolerance test (ITT), and gut leakage, which were performed 3 days prior to sacrifice according to a previous publication^24^. At sacrifice, mouse organs (livers and skin) were promptly flash-frozen in liquid nitrogen and stored at -80 °C until further use and subsequent analysis. For fecal collection, the 3 mice were separated a cage and only the feces of a mouse in the cage were collected for the microbiome analysis to avoid the impact of coprophagy (the ingestion of feces from other mice). The feces from total parts of the colon mouse were collected and combined for microbiome analysis at sacrifice.

The both of glucose and fructose levels were determined using the colorimetric assay kits (Cayman Chemical, Ann Arbor, MI, USA), while insulin levels were assessed using the mouse Ins1/insulin ELISA kit (Sigma-Aldrich, St. Louis, MO, USA). Total cholesterol and triglyceride concentrations were evaluated using the lipid quantification kit (Sigma-Aldrich), while low-density lipoprotein cholesterol (LDL) and high-density lipoprotein cholesterol (HDL) were measured using lipid profile assays (Crystal Chem Inc., Downers Grove, IL, USA). Liver damage, as indicated by serum cytokine levels, and serum alanine transaminase levels were assessed using the EnzyChrom Alanine Transaminase Assay (EALT-100; BioAssay Systems, Hayward, CA, USA) and enzyme-linked immunosorbent assays (ELISA) for mouse cytokines (Invitrogen, Carlsbad, CA, USA), respectively. The Homeostatic Model Assessment (HOMA) score was calculated using the formula: HOMA = [fasting insulin in µU/mL × fasting blood glucose in mmol/L]/ 22.5. For the oral glucose tolerance test (OGTT), mice were fasted overnight (18 h) and then orally administered glucose (2 mg/kg body weight) prior to blood glucose measurement. For the insulin tolerance test (ITT), mice fasted for 6 h were intraperitoneally administered human insulin (HumulinR, 0.75 unit/kg body weight), and blood glucose measurements were subsequently taken at 0, 15, 30, 60, 90, and 120 min. The results were calculated into the area under the curve (AUC) for both OGTT and ITT using the trapezoidal rule. Concurrently, a fluorescein isothiocyanate dextran (FITC-dextran) assay was conducted to assess gut permeability, following methods outlined in previous publications^38–40^ This procedure entailed orally administering 12.5 mg of FITC-dextran, a nonabsorbable molecule with a molecular mass of 4.4 kDa (Sigma-Aldrich), and then detecting FITC-dextran in serum using a Fluorospectrometer (NanoDrop 3300; ThermoFisher Scientific, Wilmington, DE, USA) 3 h post-administration. Serum endotoxin (LPS) levels were measured using the HEK-Blue LPS Detection kit (InvivoGen, San Diego, CA, USA).

### Intervention groups

In this 12-week study, 40 male C57BL/6 mice were allocated into five distinct groups to investigate the effects of dietary composition and Lp dfa1 supplementation on various health parameters. The groups were as follows:

- Group 1 (Control - RD): Served as the control group, receiving a regular diet with a carbohydrate: protein: fat (C: P:F) ratio of 56:13:31, representative of standard laboratory chow.
- Group 2 (High Glucose Diet - HGD): Fed a high glucose diet with an adjusted C: P:F ratio of 60:25:15, designed to simulate a high carbohydrate intake.
- Group 3 (HGD + Lp): Received the same high glucose diet as Group 2, supplemented with 1 × 10^9^ colony-forming units (CFU) of Lp dfa1 daily, to assess the probiotic’s impact on mitigating high glucose diet effects.
- Group 4 (High-carbohydrate Biscuit Diet - HBD): Consumed a high-fiber dessert diet, specifically including a high-fiber biscuit to adjust the regular diet to a C: P:F ratio of 70:9:21, aimed at evaluating the influence of dietary fiber.
- Group 5 (HBD + Lp): Given the high-carbohydrate biscuit diet with daily supplementation of 1 × 10^9^ CFU of Lp dfa1, to study the combined effect of high dietary fiber and probiotic intervention.

### Abundance of probiotics and short-chain fatty acid (SCFA) in feces

To determine the abundance of Lactiplantibacillus spp. in feces, the real-time polymerase chain reaction (PCR) was performed according to a previous protocol^41^. Briefly, the total DNAs from feces (20 mg per mouse) were extracted by a QIAamp fast DNA Stool Mini Kit (Qiagen, Hiden, Germany) following manufacturer’s instructions with the primers for variable regions of 16S rRNA gene sequence of *L. plantarum*; Lplan-vreg (forward; 5’-TTACATTTGAGTGAGTGGCGAACT-3’) and Lplan-vreg (reverse; 5’-AGGTGTTATCCCCCGCTTCT − 3’)^42^. The quantification of fecal bacteria was evaluated by real-time PCR using a QuantStudio™ Design & Analysis Software v1.4.3 and bacterial abundance was represented by cycle threshold (Ct value). For fecal SCFA, the fecal fatty acids were extracted before the analyzing by gas chromatography–mass spectrometry (GC-MS) according to previous publications^43,44^. Briefly, feces (20 mg in 500 µL normal saline) were added with 10% H2SO4 before fatty acids separation by anhydrous ether (800 mL) and centrifuged (18,000 g for 15 min). Then, the upper ether phase was mixed with 0.25 g of anhydrous Na2SO4 for 30 min, centrifuged (18,000 g for 5 min), and SCFA in the upper diethyl ether phase was determined by GC-MS using the headspace solid-phase microextraction method with an Agilent6890 GC equipped with an Agilent 5973 mass selective detector (Agilent Technologies). The dimension of the column was 0.25 mm×30 m×0.25 mm with Helium carrier gas at 13.7 ml/min. The temperature program was 10 min isothermal at 50 °C, 10 min rising to 240 °C with 15 °C per min. The injection port temperature is 200 °C while the detector port temperature is 250 °C. The mass spectrometer was operated in the electron impact mode at 70 eV with a scan range was 40–200 amu. A standard curve was obtained for the calculation of each SCFA concentration.

### Liver analysis and fecal Microbiome analysis

For liver analysis, 4 µm thick paraffin-embedded sections of liver tissue, stained with Hematoxylin and Eosin (H&E) from 10% formalin-fixed samples, were examined using the following scoring system: lobular inflammation (0–3), hepatocellular ballooning degeneration (0–2), and steatosis (0–3)^45^. The subcutaneous fat thickness was determined through direct measurement^46^. For the detection of components in the liver, liver samples were sonicated (using a High-Intensity Ultrasonic Processor, Newtown, CT, USA) in 500 µL of ice-cold PBS containing protease inhibitor Cocktail (I3786) (Sigma-Aldrich). The measured components (glucose, fructose, and lipids) were then quantified from the supernatant using the assays mentioned above. Oxidative stress was identified through the malondialdehyde (MDA) assay (Cayman Chemical Company)^47^. For the evaluation of antioxidant molecules, liver samples were sonicated in 2-(N-morpholino) ethane sulfonic acid (MES) buffer (Sigma-Aldrich) before measuring glutathione (GSH) levels (Cayman Chemical Company) from the supernatant. As for the brief fecal microbiome analysis, fecal samples (0.25 g per mouse) were processed according to a previously established protocol^48^. Metagenomic DNA extraction was performed using the QIAamp PowerFecal Pro DNA Kit (Qiagen, Hilden, Germany), with the quality of the extracted DNA assessed using the DeNovix QFX Fluorometer. The prokaryotic 16S rRNA gene at the V3V4 region was then amplified using the Qiagen QIAseq 16S/ITS Region panel (Qiagen, Hilden, Germany). Subsequently, the 16S rRNA amplicons were labeled with different sequencing adaptors using the QIAseq 16S/ITS Region Panel Sample Index PCR Reaction (Qiagen, Hilden, Germany). The resulting approximately 630 bp DNA libraries were evaluated for both quality and quantity using the QIAxcel Advanced (Qiagen, Hilden, Germany) and the DeNovix QFX Fluorometer, respectively. The Universal prokaryotic primers 341F (5’-CCTACGGNGGCWGCAG-3’) and 805R (5’-GACTACHVGGGGTATCC-3’) were utilized to amplify the V3–V4 regions of the 16 S rRNA, followed by sequencing using an Illumina Miseq600 platform (Illumina, San Diego, CA, USA) at Porcinotec Company, Nonthaburi, Thailand. The raw sequences were subjected to operational taxonomic unit (OTU) classification using Mothur’s standard operating platform procedures^49,50^. Subsequently, various bioinformatic analyses were conducted, including assessment of Good’s coverage, alpha diversity metrics (e.g., Chao index), beta diversity, Linear discriminant analysis effect size (LEfSe), and meta-stats^49,51^. The (LEfSe) method with pairwise Kruskal–Wallis and Wilcoxon tests to identify the microbial metabolic biomarker representing healthy and disease groups. This method was applied using default parameter, LDA score cutoff greater than 4, and alpha significance of 0.05. The false discovery rate for microbiome analysis was calculated using the Benjamini-Hochberg procedure in the SPSS.

### Responses of hepatocytes and enterocytes

HepG2 (hepatocyte) and Caco-2 (enterocyte) cell lines were selected for the in vitro tests due to their relevance to the potential impact of high carbohydrate content on both the gut and liver. Hence, prior to experimentation, HepG2 (HB-8065) or Caco-2 (HTB-37) cells sourced from the American Type Culture Collection (ATCC) (Manassas, VA, USA) were cultured in Dulbecco’s modified Eagle medium (DMEM) supplemented with 5.5 mM of glucose, and maintained at 37°C under 5% CO2. The cells were then seeded at a density of 1 × 10^6^ cells/well and exposed to an additional 25 mM/well of glucose, either with or without 100 µg/mL of lipopolysaccharide (LPS) derived from E. coli O26:B6 (Sigma-Aldrich). Subsequently, the supernatant cytokines were quantified utilizing the Quantikine Immunoassay method (R&D Systems, Minneapolis, MN, USA). In enterocytic experiments, gene expression of *occludin* and nuclear factor kappa B (*NF-κB*) in relative to glyceraldehyde-3-phosphate dehydrogenase *(GAPDH*) (a house keeping gene) was evaluated using the 2^-ΔΔCp^ method^52^ with the following primers; *occludin*, forward 5’- CCAATGTCGAGGAGTGGG-3’, reverse 5’-CGCTGCTGTAACGAGGCT-3’; *NF-κB*, forward 5’- AGCACAGATACCACCAAGACC − 3’, reverse 3’-GGGCACGATTGTCAAAGAT-5’; *MUC2*, forward 5’-CCTGCCGACACCTGCTGCAA-3’, reverse 5’- ACACCAGTAGAAGGGACAGCACCT − 3’; *GAPDH*, forward 5’-GCACCGTCAAGGCTGAGAAC-3’, reverse 5’-ATGGTGGTGAAGACGCCAGT-3’.

In parallel, transepithelial electrical resistance (TEER) measurements were performed using Caco-2 cells seeded at a density of 5 × 10^4 cells per well on the upper compartment of a 24-well Boyden chamber trans-well plate. The cells were cultured in DMEM-high glucose supplemented with 20% Fetal Bovine Serum (FBS), 1% HEPES, 1% sodium pyruvate, and 1.3% Penicillin/Streptomycin for 15 days to establish a confluent monolayer. Subsequently, glucose (25 mM) with or without 1 µg/mL of lipopolysaccharide (LPS) from *E. coli* O26:B6 (Sigma-Aldrich) was added and incubated at 37 °C under 5% CO2 before TEER measurement. TEER was measured using an epithelial volt-ohm meter (EVOM-2, World Precision Instruments, Sarasota, FL, USA) with electrodes positioned in the supernatant at both the basolateral and apical chambers. The TEER value in ohm (Ω) x cm2 of the media culture without cells acted as a blank and was subtracted from all measurements. A lower TEER value indicates greater permeability damage of Caco-2 cells.

### Statistical analysis

The data were presented as mean ± standard error (SE) and analyzed utilizing one-way analysis of variance (ANOVA) followed by Tukey’s post-hoc analysis. The false discovery rate for microbiome analysis was calculated using the Benjamini-Hochberg procedure in the SPSS 11.5 software (SPSS, IL, USA) and the adjusted p value (q value) less than 0.05 was used as an accepted hypothesis. Statistical analyses were performed using SPSS and GraphPad Prism version 10 software (La Jolla, CA, USA). A p-value less than 0.05 was deemed statistically significant.

## Results

### Mitigation of obesity and prediabetes by Lp dfa1 in mice on HGD and HBD

**Fig. 1:**
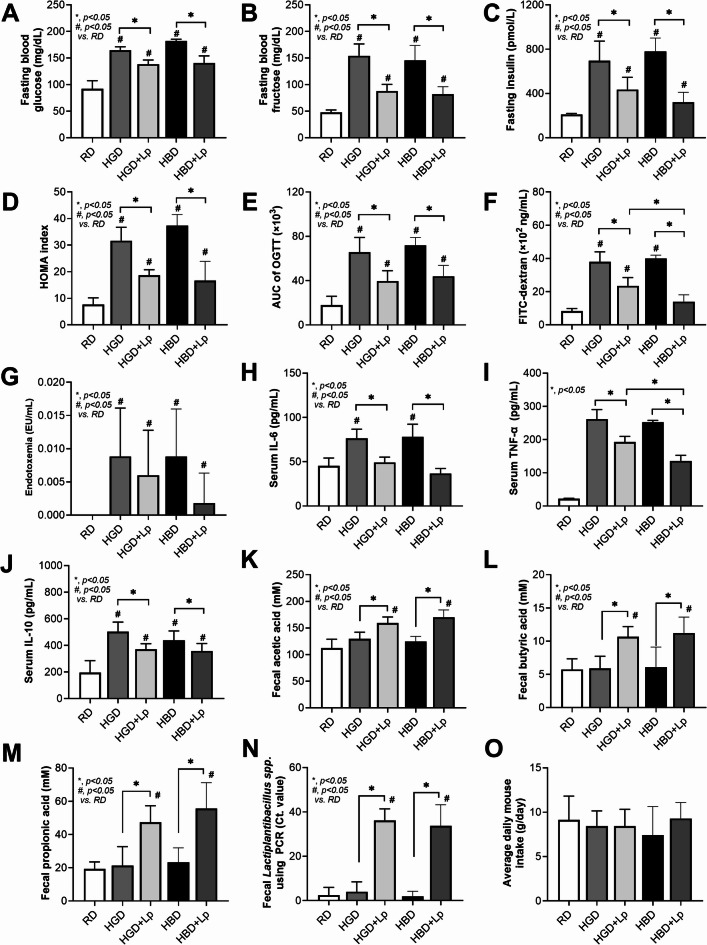
Metabolic and inflammatory responses to dietary interventions, including regular diet (RD), high glucose diet (HGD), or high-carbohydrate biscuit diet (HBD), with or without *Lactiplantibacillus plantarum* (Lp) dfa1: Fasting glucose, fructose, and insulin levels (A-C), Homeostatic Model Assessment for Insulin Resistance (HOMA-IR index) (D), Oral glucose tolerance test (OGTT) with the area under the curve (AUC) (E), Gut permeability assessed by FITC-dextran assay (F), Serum LPS (endotoxemia) (G), Serum cytokines (IL-6, TNF-α, and IL-10) (H-J), and Fecal short-chain fatty acid (acetic acid, butyric acid, and propionic acid) (K-M), Fecal abundance of *Lactiplantibacillus spp.* (N), and Daily food intake (O).

**Fig. 2:**
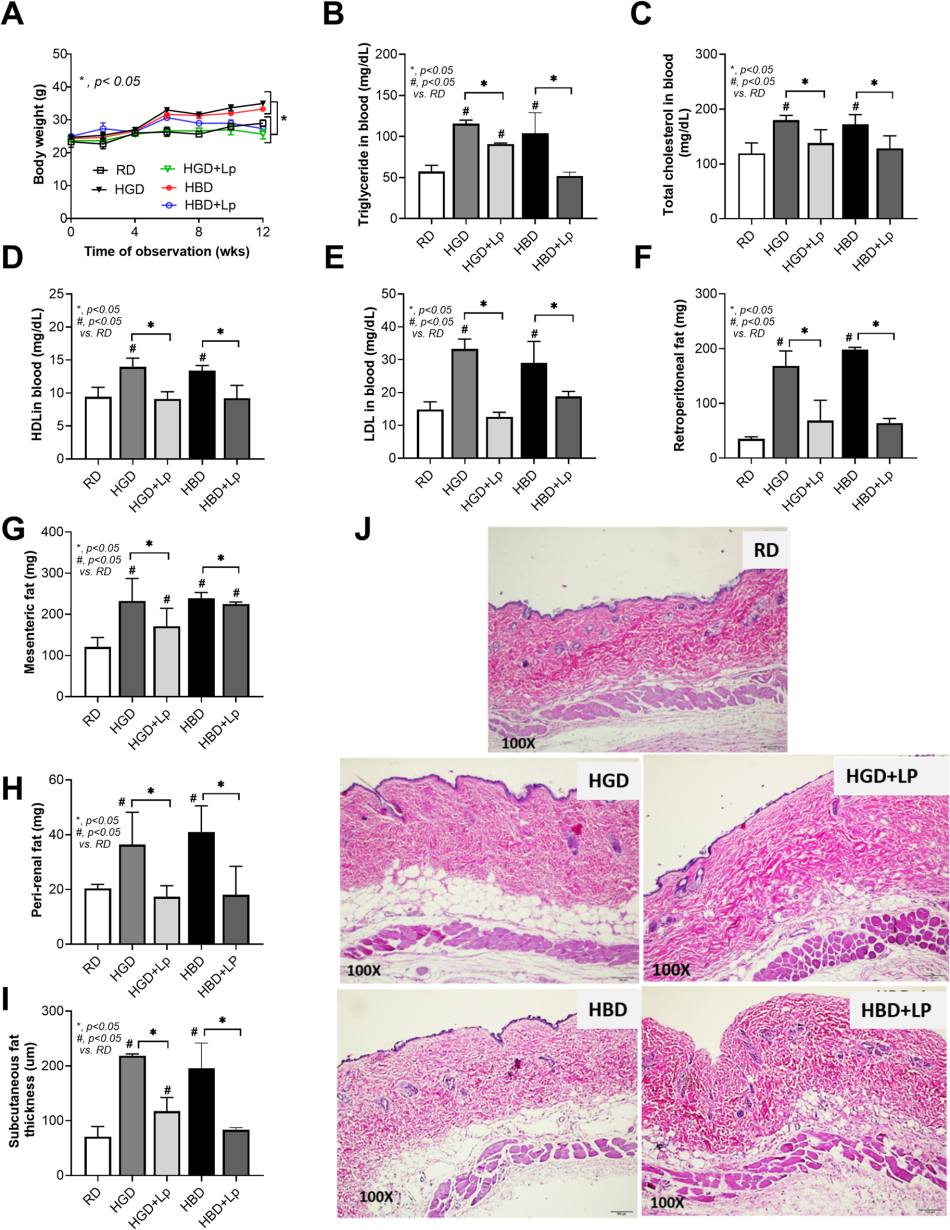
Obesity-related outcomes in mice under different dietary conditions, including regular diet (RD), high glucose diet (HGD), or high-carbohydrate biscuit diet (HBD), with or without *Lactiplantibacillus plantarum* (Lp) dfa1: Body weight at 12 weeks (A), Serum triglyceride, total cholesterol, high-density lipoprotein (HDL), and low-density lipoprotein (LDL) (B-E), Visceral and subcutaneous fat deposition (F-J). Representative figures of subcutaneous fat thickness were captured using an original magnification of 100x are demonstrated.

**Fig. 3:**
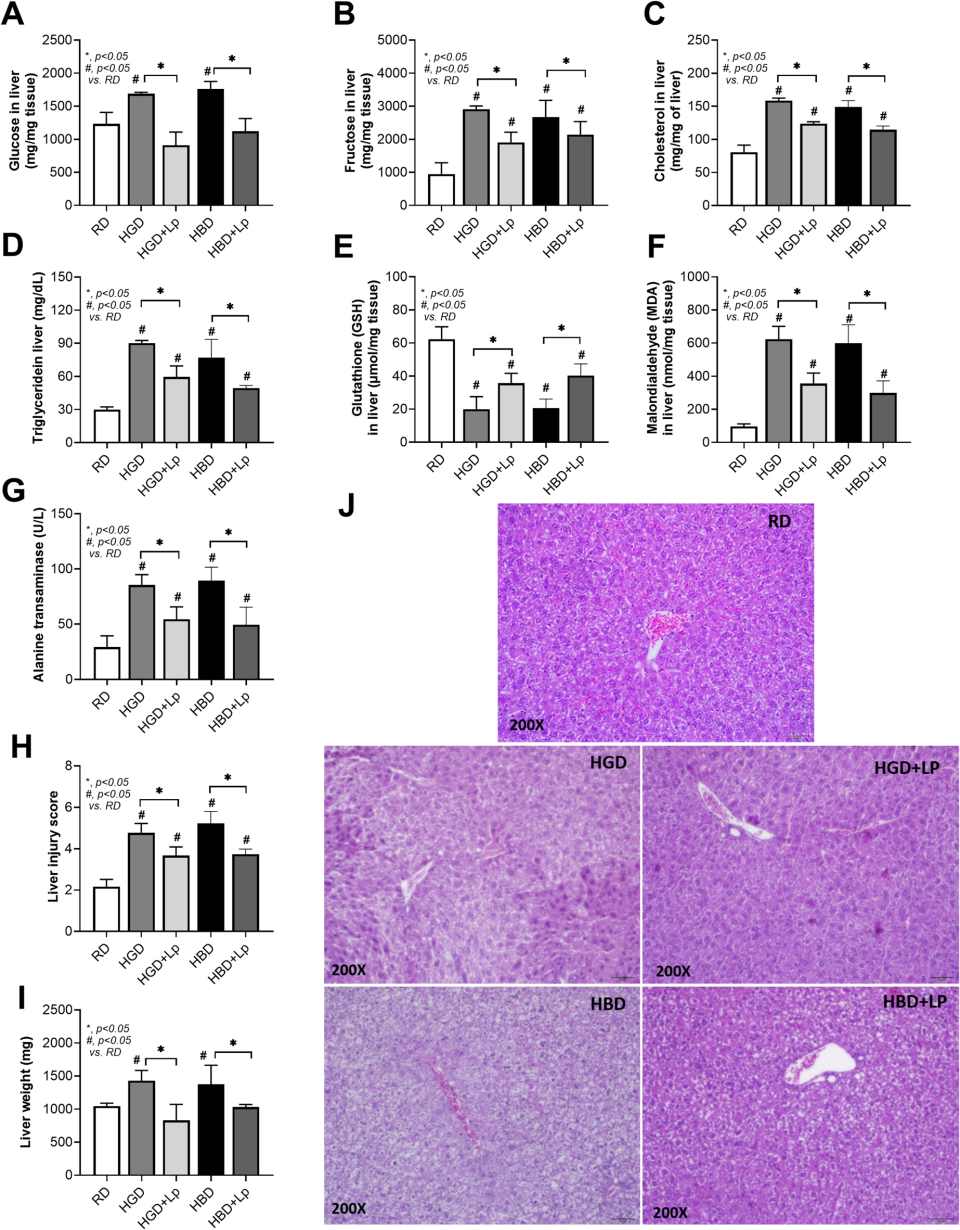
Liver injury and oxidative stress in response to different dietary conditions, including regular diet (RD), high glucose diet (HGD), or high-carbohydrate biscuit diet (HBD), with or without *Lactiplantibacillus plantarum* (Lp) dfa1: Liver content of glucose, fructose, cholesterol, and triglycerides (A-D), Oxidative stress (MDA) and antioxidant (GSH) levels (E-F), and liver injury parameters including serum alanine transaminase (ALT) levels, histological injury score for steatosis and inflammation, and liver weight with representative histological images (G-J) are demonstrated.

Both HGD and HBD diets induced a prediabetic state, demonstrated by altered fasting blood glucose, fructose, insulin levels, insulin resistance (HOMA-IR), and impaired OGTT (Fig. 1A-E). The negative impact on intestinal health was also parallel, with both diets increasing gut permeability (FITC-dextran assay) and serum endotoxin levels (serum lipopolysaccharide) (Fig. 1F, G), leading to higher systemic inflammation (elevated IL-6, TNF-α, and IL-10 levels) in comparison to the RD group (Fig. 1H-J). Also, the lower serum cytokines in probiotic-administered mice may be due to the increased fecal SCFA (acetate, butyrate, and propionate) (Fig. 1K-M). Notably, the elevated abundance of Lp dfa1 was also supported by increased abundance of probiotics in feces as determined by PCR from feces (Fig. 1N), while the daily intake of mice in all groups was not different (Fig. 1O). Administration of both HGD and HBD resulted in comparable elevations in obesity markers, evidenced by increases in body weight by the 12th week, changes in serum lipid profiles (including triglycerides, total cholesterol, HDL, and LDL levels), and visceral fat deposition across several sites such as the retroperitoneum, mesentery, perirenal, and subcutaneous areas (Fig. 2A-J). Liver assessments further showed no difference between the two diets in exacerbating liver damage, as indicated by the liver’s biochemical composition (glucose, fructose, cholesterol, and triglycerides), oxidative stress markers (glutathione for antioxidants, malondialdehyde for pro-oxidant, liver enzyme activity (alanine transaminase), liver weight, and histological injury scores (Fig. 3A-J).

However, the introduction of Lp dfa1 led to a marked improvement across all measured parameters of obesity, prediabetes, and liver health in both HGD and HBD groups (Figs. 1, 2 and 3), underscoring its protective effect against dietary-induced metabolic impairments. Specifically, Lp dfa1 supplementation was effective in strengthening intestinal integrity, reducing gut permeability, and mitigating the severity of endotoxemia, which, in turn, decreased systemic inflammatory cytokine levels (Fig. 1F-J). Because (i) Gram-negative bacteria in the gut is a source of endotoxin (LPS)^10^ which could enter blood circulation (obesity-induced endotoxemia)^14^ and (ii) gut dysbiosis causes gut barrier defect is well-known^48,53,54^ the effect of Lp dfa1 on gut barrier strengthening (serum FITC-dextran assay) might be, partly, due to the impact on gut dysbiosis.

### Gut microbiota altertion and the impact of Lp dfa1

The fecal microbiome analysis revealed distinct patterns of bacterial abundance across phyla, classes, orders, families, and genera between control mice and those with carbohydrate-induced obesity (Supplement Fig. 1A-F). Notably, the alpha diversity, as measured by the Chao1 index, showed only a mild difference, with the highest diversity observed in mice on the HGD + Lp diet, while the Shannon index remained unaffected (Supplement Fig. 1G-H). Non-metric multidimensional scaling (NMDS) illustrated the group-specific clustering of microbiome compositions, indicating distinct microbial communities associated with each dietary regimen despite sample collection from various cages (Supplement Fig. 1I).

**Fig. 4:**
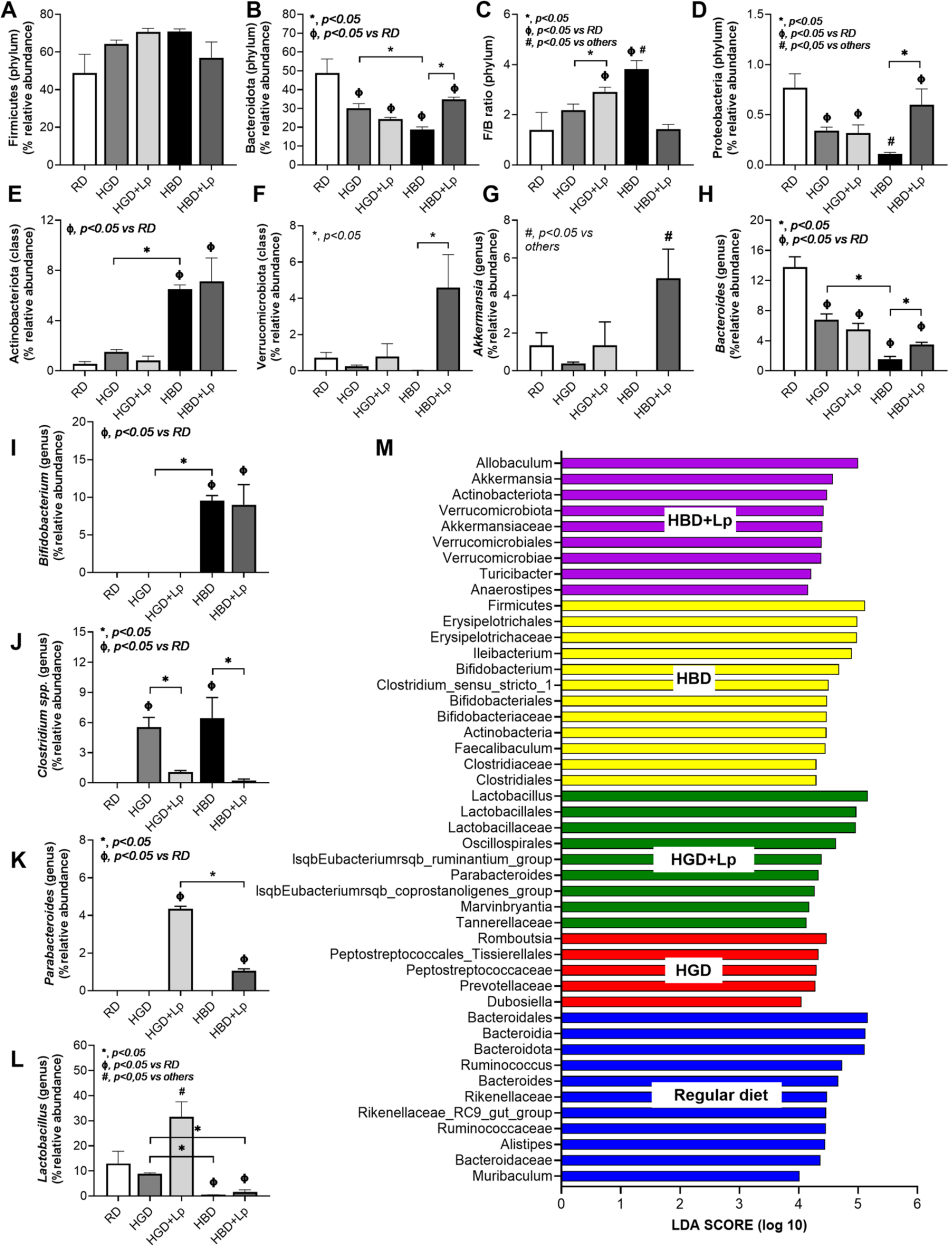
Taxonomic changes in gut microbiota composition (in the graph presentation) in mice with regular diet (RD), high glucose diet (HGD), or high-carbohydrate biscuit diet (HBD), with or without *Lactiplantibacillus plantarum* (Lp) dfa1: Relative abundance of key bacterial in phylum level, including *Firmicutes*, *Bacteroidota*, *Firmicutes/Bacteroidota* ration (F/B ratio), and *Proteobacteria* (A-D), in class level (*Actinobacteriota* and *Verrucomicrobiota*) (E, F), and in genus level (*Akkermansia spp.*, *Bacteroides spp.*, *Bifidobacterium spp.*, *Clostridium spp.*, *Parabacteroides spp.*, and *Lactobacillus spp.*) (G-L) together with the Linear discriminant analysis Effect Size (LEfSe) discriminant features (the representative bacteria) among experimental groups (M) are demonstrated.

Comparative analysis underscored a nuanced shift in microbial populations between the RD and HGD groups, with a notably closer NMDS proximity between these groups than others (Supplement Fig. 1I). Detailed dysbiosis patterns, when comparing HGD and RD, included reduced Bacteroidota and Proteobacteria phyla, diminished *Bacteroides* spp., and an increase in *Clostridium* spp. (genus) (Fig. 4A-L). The HBD group, relative to RD, exhibited lower Bacteroidota, an increased Firmicutes/Bacteroides (F/B) ratio, decreased Proteobacteria, elevated Actinobacteriota at the class level, and higher *Bifidobacterium* spp. and *Clostridium* spp. at the genus level (Fig. 4A-L). Comparing HBD to HGD, the findings were similar in terms of lower Bacteroidota and higher F/B ratio, with additional distinctions at the genus level, including lower *Bacteroides and higher Bifidobacterium* and *Lactobacillus* (Fig. 4A-L).

Lp dfa1 supplementation in HGD diets modulated the microbiota by increasing F/B ratio, decreasing *Clostridial* spp., and increasing *Lactobacilli* populations (Fig. 4A-L). In HBD diets, Lp dfa1 supplementation led to increases increases in Bacteroides (phylum level), Verrucomycobiota, *Akkermansia* spp., *Bacteroides* spp., *Parabacteroides* spp., alongside a reduction in the F/B ratio and *Clostridial* spp. (Fig. 4A-L). With LEfSe analysis identifying unique bacterial signatures for each group: control (dominated by Bacteroides), HGD (characterized by *Rombutsia*), and HBD (predominantly *Firmicutes*). Notably, Lp dfa1 shifted the bacterial landscape in HBD towards *Allobaculum*^55^ and *Akkermansia*, known for their beneficial roles in obesity^56^ while in HGD, *Lactobacilli* became more prevalent (Fig. 4M). Additionally, heat map analysis further delineated genus-level bacterial abundance changes and the Cladogram showed the evolutionary relationships among bacteria in our experimental groups (Supplement Fig. 2A, B).

### Additive cell damage from lipopolysaccharide and glucose and attenuation by probiotioc supernatant

**Fig. 5:**
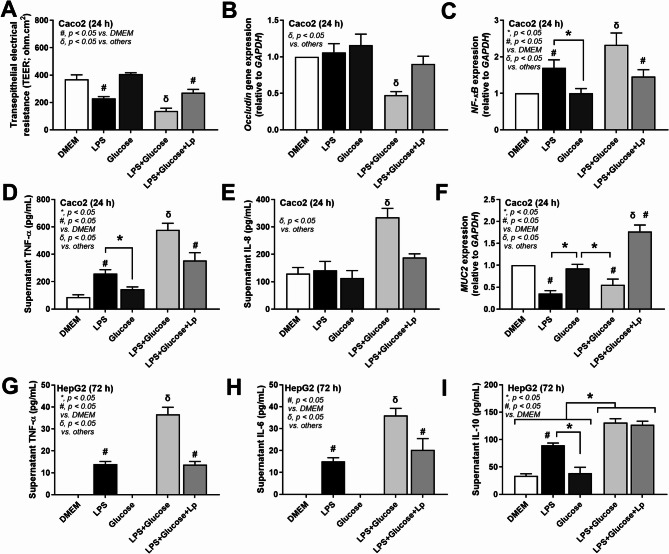
In vitro effects of the activation by media control (DMEM), lipopolysaccharide (LPS), glucose, glucose with LPS (LPS + Glucose), or LPS + Glucose with supernatant of probiotics (LPS + Glucose + Lp) in enterocytes and hepatocytes: Transepithelial electrical resistance (TEER) measurements in Caco-2 cells (A), *Occludin* (a tight junction molecule), nuclear factor kappa B (*NF-κB*), mucin (*MUC2*) gene expression, cytokine release (IL-8, TNF-α) (B-F), HepG2 cytokine responses to glucose + LPS with and without probiotic-conditioned media (G-I).

Enterocytes naturally resist LPS, a prevalent microbial molecule from Gram-negative bacteria found in feces^57^. Yet, the combination of high dietary glucose levels and gut LPS concentration may exert additive pro-inflammatory effects. Specifically, high concentration of LPS, unlike glucose, compromised enterocyte permeability, as reflected by reduced transepithelial electrical resistance (TEER) measurements (Fig. 5A). An additive detrimental effect was observed with LPS and glucose-co-exposure, shown by decreased TEER, reduced *occludin* expression (a tight junction protein), and increased *NF-κB* expression alongside elevated supernatant cytokine levels and decreased mucin compared to LPS alone in enterocytes (Caco-2 cells) (Fig. 5A-F). The diminished TEER and heightened *NF-κB* and cytokine levels without *occludin* alteration post-LPS challenge suggest a lesser sensitivity of occludin as a damage indicator relative to other metrics (Fig. 5A-F). Despite enterocyte resistance against LPS, the selected dose of LPS can damage enterocytes as indicated here by all of these parameters except for occludin and supernatant IL-8 (Fig. 5A-F). With the probiotic condition media, all of these parameters were attenuated when compared with non-probiotic activation (Fig. 5A-F).

The introduction of probiotic-conditioned media ameliorated these effects showcasing a protective role against LPS and glucose-inducing damage (Fig. 5A-F). This finding underscores the previously documented glucose-driven enterocyte integrity compromise^58^ and highlights the beneficial impact of probiotics in maintaining gut health.

In hepatocytes, glucose alone did not induce inflammatory responses. However, the combination of LPS and glucose elicited more severe injury, as indicated by increased supernatant cytokine levels, compared to LPS exposure alone (Fig. 5G-I). Probiotic-conditioned media reduced TNF-α and IL-6 levels, though not IL-10, in response to LPS and glucose, indicating that probiotics exert similar anti-inflammatory effects on both enterocytes and hepatocytes (Fig. 5G-I).

## Discussion

Both high glucose diet (HGD) and high-carbohydrate biscuit diet (HBD) similarly induced severity of obesity and liver injury with different alteration in fecal microbiome analysis and *L. plantarum* dfa1 (Lp dfa1) attenuated obesity, partly through an anti-inflammatory effect and microbiome alteration.

### Similar impact of glucose from both diets in obesity but not in fecal Microbiome analysis

The diets provided, HGD and HBD, with their distinct carbohydrate: protein: fat (C: P:F) ratios of 60:25:15 and 70:9:21 respectively, both led to obesity and prediabetes, despite having the same calculated energy content (3.04 kcal/g) as the regular mouse diet (RD; C:P: F of 56:13:31). Despite the lower fat ratio in HGD and HBD compared with RD, obesity in the experimental mice was a result of the sugars but not from the fat component of the diet. Interestingly, both glucose in HGD and the sugar components of HBD (sucrose and, possibly fructose from the pineapple ingredient) could induce indistinguishable obesity and prediabetes in mice, as indicated by several parameters, such as body weight, lipid profiles, fasting plasma glucose and OGTT. In the intestine, glucose primarily enters the bloodstream through the Na+/glucose cotransporter 1 (SGLT1), followed by GLUT2^59^, Conversely, fructose is absorbed via glucose transporters 5 (GLUT 5) and then diffuses into the bloodstream primarily through GLUT2 (and to a lesser extent, GLUT 5), independent of sodium absorption and ATP hydrolysis. This process leads to significant fructose uptake by the liver^60,61^. Dietary sucrose is digested by sucrase and splits sucrose molecule into glucose and fructose. The fructose absorbed in the small intestines and liver is usually altered into glucose through a well-known process called fructose-induced gluconeogenesis in the liver^62,63^ which might be responsible for the increased blood glucose levels after fructose absorption. In contrast, the conversion from glucose into fructose in the body (endogenous fructose production) is also possible via the activation of the polyol pathway, as demonstrated in metabolic syndrome and renal disease^64,65^. The glucose-fructose interchangeable supports the importance of both sugars in the pathogenesis of metabolic syndrome^66–68^ and a similar ration of carbohydrate in HGD and HBD at 60 and 70%, respectively, might be the major factor inducing obesity and prediabetes in our mice. Because the percentage of fructose in HBD might not obviously high, there was similar impacts of HGD (glucose alone) and HBD (glucose with fructose). Nevertheless, we previously report the difference between high glucose versus fructose administration^24^ as there was more prominent hepatic adverse effect (non-alcoholic fatty liver)^67^ with less severe enterocyte toxicity^58^ from fructose than glucose.

There was a well-known data on gut dysbiosis from the selective growth of some group of bacteria with different carbohydrate in the gut content^69^; for example, the responses against sucrose of *Bacillus atrophaeus* and *Acinetobacter nectaris* are different^70^. Also, different carbohydrate might induce different effects on some organisms. As such, the presence of glucose enhances the sensitivity toward bacteriocin of *Listeria monocytogenes*, while sucrose increased the resistance^71^. Here, there was some differences in gut dysbiosis between both diets which might, at least in part, due to the fruit-derived soluble fiber in HBD because the major component of both HGD and HBD should be glucose. As such, the fruit-derived soluble fiber might partly be responsible for the elevation of Firmicutes/ Bacteroides ration (F/B ration) as the ability to digest plant biomass (cellulose) of Firmicutes are possibly more prominent than Bacteroides, especially at an initial stage of the decomposition^72^. As such, several Firmicutes, including Bacillus, Clostridium, Rumminococcus, and Acetivibrio, and a few bacteria in other phylum; such as Bacteroides (*Bacteriodes succinogenes*), Actinomycetota (*Cellulomonas*), and Proteobacteria (*Alteromonas*) are cellulolytic bacteria (Fig. 4B-D)^73^. Here, the elevated Actinobacteriota in HBD compared with HGD might be a result from the fruit-derived soluble fiber in HBD (Fig. 4B). Hence, the dessert containing fruit-derived soluble fiber might have some benefit on the gut microbiota compared with the dessert with pure carbohydrate. However, the similar severity of obesity and prediabetes between HGD and HBD, despite some difference in fecal microbiome, suggested a less impact of gut microbiome on the clinical syndrome. Although the fruit-derived soluble fiber has some helpful impacts on the selection of beneficial bacteria, the adverse effect on glucose on enterocytes might overcome benefits from fruit-derived soluble fiber. Indeed, leaky gut (FITC-dextran assay) and endotoxemia were not different between HGD and HBD.

### Lactiplantibacillus plantarum dfa1, microbiome, and anti-inflammatory impact

The attenuation of obesity by probiotics through several mechanisms, including more efficient energy utilization, promotion of intestinal hormones, and reduction in lipid absorption within the host^14,54,74,75^ are well known. Also, the effectiveness of *L. plantarum* dfa1 against intestinal damages from lipid are also mentioned^35^. Here, Lp dfa1 similarly attenuated all aspects of obesity from both HGD and HBD with some differences in fecal microbiota from both diets. As such, the growth of *Lactiplantibacillus* in feces of mice with high glucose seems to be better than the mice with HBD (Fig. 4L); however, the administered *Lactiplantibacillus* elevated several beneficial bacteria, especially *Akkermansia* (Fig. 4G). As such, the anti-obesity impacts of *Akkermansia* is well-known through several mechanisms, including the improved energy metabolism and energy consumption of the host^25^. Although the growth of the administered *Lactiplantibacillus* in HBD seems to be lower than in HGD (Fig. 4L; HGD + Lp vs. HBD + Lp), the anti-obesity effect of probiotics in HBD was similar to HGD. This might be due to the compensatory elevation of the increased *Akkermansia*. Notably, *Bifidobacterium*, another beneficial bacterium^76^ in HBD was also higher than other groups which might also be due to the fruit-derived soluble fiber supporting the well-digestive property on fiber-riches diet by this bacterium^77^. Indeed, *Lactobacillus*,* Bifidobacterium*,* Clostridium*, and *Akkermansia* are beneficial bacteria against insulin resistance and obesity though several possibly mechanisms, including reduced carbohydrate absorption, improved energy utilization, facilitated some intestinal enzymes, and anti-inflammation^78,79^. Hence, the beneficial effects of *L. plantarum* dfa1 might be due to an indirect impact (the elevation of other beneficial bacteria in the gut) and a direct effect from an improved energy utilization and anti-inflammation^24^. For the enhanced *Akkermansia* effect of Lp when administered with HBD (HBD + Lp) but not HGD + Lp, we hypothesize that this is partly due to the activation of mucin production by some molecules excreted by Lp as the Lp condition media upregulated MUC2 gene in Caco2 enterocytes (Fig. 5F). The elevated mucin production may enhance the growth of *Akkermansia* (a mucin-degrading bacterium)^80^. Because of the lower abundance of *Akkermansia* in HGD + Lp than in HBD + Lp, some gut bacteria that are elevated by HBD; for example, Actinobacteriota and Bifidobacterium (Fig. 4E and I), which consist of some bacteria with mucin-enhancing properties^81,82^ might also be important. More studies are interesting.

For the carbohydrate-induced pro-inflammation, impacts of hyperglycemia-induced cell injury distribute to all cells in the body, despite the main sugar absorption site at the small intestine^83^. Unsurprisingly, hyperglycemia induces hyper-inflammatory activation, liver injury, and the enterocyte damage (throughout the intestinal tract) as indicated by gut permeability defect (FITC-dextran assay and metabolic endotoxemia) supporting previous publications^24,84^. Here, the activation of glucose in a high dose additively elevated LPS-induced pro-inflammation and induced permeability defect (TEER) in Caco-2 enterocytes which were compatible with leaky gut and increased pro-inflammatory cytokines in mice. Likewise, the pro-inflammatory synergy between glucose and LPS was also demonstrated in HepG2 hepatocytes from the elevated supernatant TNF-α and IL-6. Despite the additive hyper-inflammation of glucose plus LPS compared with LPS alone, condition media of the probiotics attenuate the inflammation in both enterocyte and hepatocytes. Indeed, the exopolysaccharides are one of the possible anti-inflammatory molecules in the condition media that are secreted from Lp dfa1^35^. Although the supernatant of Lp dfa1 can attenuate hepatocyte inflammation in vitro, these results could not explain the beneficial impact on mouse liver because the molecular weight of the exopolysaccharide might be too high to pass through the gut barrier to be delivered to the liver. However, the probiotic molecules that equal to or smaller than LPS (approximately molecular weight at 30–60 kDa)^10,85^ might possibly be delivered to the liver to demonstrate beneficial effects. Despite a necessity for the more detail mechanistic studies, our proof-of-concept data demonstrate a synergy between LPS and glucose on the injury in both enterocytes and hepatocytes that was attenuated by the probiotics. More studies are interesting.

There were several limitations due to the study’s “proof of concept” characteristics, particularly with regard to the mechanical interpretation of the observed data. It would be interesting to see further research on metagenomic, metabolomic, and functional microbiota studies. Although our data support the use of probiotics for a prevention of high carbohydrate-induced prediabetes, more research on these subjects is necessary for the upcoming clinical translation.

## Conclusions

Both glucose alone (HGD) and glucose with fruit-derived soluble fiber (HBD) induced similar damages on the intestinal integrity (leaky gut) and obesity-related parameters; however, with different impact on gut microbiota as higher Firmicutes/ Bacteroides ration (F/B), Actinobaceriota, and Bifidobacterium in HBD and higher *Lactiplantibacillus* in HGD. Then, probiotics attenuated condition in both diets, partly through increased *Akkermansia* in HBD and elevated *Lactiplantibacillus* in HGD, with other non-microbiome related effects. The additive pro-inflammatory effect of LPS with glucose was attenuated in enterocytes and hepatocytes supported an anti-inflammation of the probiotics. Hence, we encourage the use of probiotics for the prevention of carbohydrate-induced obesity.

## Electronic supplementary material

Below is the link to the electronic supplementary material.

Supplementary Material 1

Supplementary Material 2

Supplementary Material 3

Supplementary Material 4

Supplementary Material 5

## Data Availability

Data is provided within the manuscript or supplementary information files.

## References

[1] Allison, D. B., Fontaine, K. R., Manson, J. E., Stevens, J. & VanItallie, T. B. Annual deaths attributable to obesity in the united States. *Jama* **282**, 1530–1538. 10.1001/jama.282.16.1530 (1999). 10546692 10.1001/jama.282.16.1530

[2] Alberti, K. G. et al. Harmonizing the metabolic syndrome: a joint interim statement of the international diabetes federation task force on epidemiology and prevention; National heart, lung, and blood institute; American heart association; world heart federation; international atherosclerosis society; and international association for the study of obesity. *Circulation* **120**, 1640–1645. 10.1161/circulationaha.109.192644 (2009). 19805654 10.1161/CIRCULATIONAHA.109.192644

[3] Pasmans, K., Meex, R. C. R., van Loon, L. J. C. & Blaak, E. E. Nutritional strategies to attenuate postprandial glycemic response. *Obes. Rev.* **23**, e13486. 10.1111/obr.13486 (2022). 35686720 10.1111/obr.13486 PMC9541715

[4] Kolyva, A. S. et al. The role of obesity in the immune response during sepsis. *Nutr. Diabetes*. **4**, e137. 10.1038/nutd.2014.34 (2014). 25244356 10.1038/nutd.2014.34 PMC4183975

[5] Singer, G., Stokes, K. Y., Terao, S. & Granger, D. N. Sepsis-induced intestinal microvascular and inflammatory responses in obese mice. *Shock* **31**, 275–279. 10.1097/SHK.0b013e3181834ab3 (2009). 18665045 10.1097/SHK.0b013e3181834ab3

[6] Frühbeck, G., Catalán, V., Rodríguez, A. & Gómez-Ambrosi, J. Adiponectin-leptin ratio: a promising index to estimate adipose tissue dysfunction. Relation with obesity-associated cardiometabolic risk. *Adipocyte* **7**, 57–62. 10.1080/21623945.2017.1402151 (2018). 29205099 10.1080/21623945.2017.1402151 PMC5915018

[7] Jaroonwitchawan, T. et al. Dysregulation of lipid metabolism in macrophages is responsible for severe endotoxin tolerance in FcgRIIB-Deficient lupus mice. *Front. Immunol.* **11**, 959. 10.3389/fimmu.2020.00959 (2020). 32582149 10.3389/fimmu.2020.00959 PMC7296175

[8] McArdle, M. A., Finucane, O. M., Connaughton, R. M., McMorrow, A. M. & Roche, H. M. Mechanisms of obesity-induced inflammation and insulin resistance: insights into the emerging role of nutritional strategies. *Front. Endocrinol. (Lausanne)*. **4**, 52. 10.3389/fendo.2013.00052 (2013). 23675368 10.3389/fendo.2013.00052 PMC3650620

[9] Ross, P. A., Newth, C. J., Leung, D., Wetzel, R. C. & Khemani, R. G. Obesity and mortality risk in critically ill children. *Pediatrics* **137**, e20152035. 10.1542/peds.2015-2035 (2016). 26908670 10.1542/peds.2015-2035

[10] Amornphimoltham, P., Yuen, P. S. T., Star, R. A. & Leelahavanichkul, A. Gut leakage of Fungal-Derived inflammatory mediators: part of a Gut-Liver-Kidney Axis in bacterial Sepsis. *Dig. Dis. Sci.* **64**, 2416–2428. 10.1007/s10620-019-05581-y (2019). 30863955 10.1007/s10620-019-05581-y

[11] Mkumbuzi, L., Mfengu, M. M. O., Engwa, G. A. & Sewani-Rusike, C. R. Insulin resistance is associated with gut permeability without the direct influence of obesity in young adults. *Diabetes Metab. Syndr. Obes.* **13**, 2997–3008. 10.2147/dmso.S256864 (2020). 32922055 10.2147/DMSO.S256864 PMC7457818

[12] Hu, R. et al. New insights into the links between anti-diabetes drugs and gut microbiota. *Endocr. Connect.* **10**, R36–r42. 10.1530/ec-20-0431 (2021). 33338029 10.1530/EC-20-0431 PMC7923145

[13] Udompornpitak, K. et al. Obesity exacerbates lupus activity in Fc gamma receptor IIb deficient lupus mice partly through saturated fatty Acid-Induced gut barrier defect and systemic inflammation. *J. Innate Immun.* **15**, 240–261. 10.1159/000526206 (2023). 36219976 10.1159/000526206 PMC10643905

[14] Pitocco, D. et al. The role of gut microbiota in mediating obesity and diabetes mellitus. *Eur. Rev. Med. Pharmacol. Sci.* **24**, 1548–1562. 10.26355/eurrev_202002_20213 (2020). 32096204 10.26355/eurrev_202002_20213

[15] Eppensteiner, J. et al. Damage- and pathogen-associated molecular patterns play differential roles in late mortality after critical illness. *JCI Insight*. **4**, 253. 10.1172/jci.insight.127925 (2019). 10.1172/jci.insight.127925 PMC6777836 31434802

[16] Jamar, G., Ribeiro, D. A. & Pisani, L. P. High-fat or high-sugar diets as trigger inflammation in the microbiota-gut-brain axis. *Crit. Rev. Food Sci. Nutr.* **61**, 836–854. 10.1080/10408398.2020.1747046 (2021). 32267169 10.1080/10408398.2020.1747046

[17] Heisel, T. et al. High-Fat diet changes fungal microbiomes and interkingdom relationships in the murine gut. *mSphere* **2**, 2. 10.1128/mSphere.00351-17 (2017). 10.1128/mSphere.00351-17 PMC5636226 29034327

[18] Bhayani, J. et al. Carbohydrate metabolism in bacteria: alternative specificities in ADP-Glucose pyrophosphorylases open novel metabolic scenarios and biotechnological tools. *Front. Microbiol.* **13**, 867384. 10.3389/fmicb.2022.867384 (2022). 35572620 10.3389/fmicb.2022.867384 PMC9093745

[19] Cheng, W. L. et al. Sugar Fructose triggers gut dysbiosis and metabolic inflammation with cardiac arrhythmogenesis. *Biomedicines* **9**, 745. 10.3390/biomedicines9070728 (2021). 10.3390/biomedicines9070728 PMC8301417 34201938

[20] Murphy, E. A., Velazquez, K. T. & Herbert, K. M. Influence of high-fat diet on gut microbiota: a driving force for chronic disease risk. *Curr. Opin. Clin. Nutr. Metab. Care*. **18**, 515–520. 10.1097/mco.0000000000000209 (2015). 26154278 10.1097/MCO.0000000000000209 PMC4578152

[21] Leelahavanichkul, A. et al. Gastrointestinal leakage detected by serum (1→3)-β-D-Glucan in mouse models and a pilot study in patients with Sepsis. *Shock* **46**, 506–518. 10.1097/shk.0000000000000645 (2016). 27172153 10.1097/SHK.0000000000000645

[22] Bray, G. A., Nielsen, S. J. & Popkin, B. M. Consumption of high-fructose corn syrup in beverages May play a role in the epidemic of obesity. *Am. J. Clin. Nutr.* **79**, 537–543. 10.1093/ajcn/79.4.537 (2004). 15051594 10.1093/ajcn/79.4.537

[23] Softic, S. et al. Divergent effects of glucose and Fructose on hepatic lipogenesis and insulin signaling. *J. Clin. Invest.* **127**, 4059–4074. 10.1172/jci94585 (2017). 28972537 10.1172/JCI94585 PMC5663363

[24] Ondee, T. et al. High Fructose causes more prominent liver steatohepatitis with leaky gut similar to high glucose administration in mice and Attenuation by Lactiplantibacillus plantarum dfa1. *Nutrients* **15**, 23. 10.3390/nu15061462 (2023). 10.3390/nu15061462 PMC10056651 36986190

[25] Ondee, T. et al. Lactobacillus acidophilus LA5 improves saturated fat-induced obesity mouse model through the enhanced intestinal Akkermansia muciniphila. *Sci. Rep.* **11**, 6367. 10.1038/s41598-021-85449-2 (2021). 33737543 10.1038/s41598-021-85449-2 PMC7973717

[26] Parnell, J. A. & Reimer, R. A. Prebiotic fiber modulation of the gut microbiota improves risk factors for obesity and the metabolic syndrome. *Gut Microbes*. **3**, 29–34. 10.4161/gmic.19246 (2012). 22555633 10.4161/gmic.19246 PMC3827018

[27] Scaldaferri, F. et al. Gut microbial flora, prebiotics, and probiotics in IBD: their current usage and utility. *Biomed. Res. Int.* **2013**, 435268. 10.1155/2013/435268 (2013). 10.1155/2013/435268 PMC3749555 23991417

[28] Hager, C. L. & Ghannoum, M. A. The mycobiome: role in health and disease, and as a potential probiotic target in Gastrointestinal disease. *Dig. Liver Dis.* **49**, 1171–1176. 10.1016/j.dld.2017.08.025 (2017). 28988727 10.1016/j.dld.2017.08.025

[29] Zuo, T. & Ng, S. C. The gut microbiota in the pathogenesis and therapeutics of inflammatory bowel disease. *Front. Microbiol.* **9**, 2247. 10.3389/fmicb.2018.02247 (2018). 30319571 10.3389/fmicb.2018.02247 PMC6167487

[30] Kathrani, A., Larsen, J. A., Kass, P. H. & Fascetti, A. J. Effect of short-term probiotic Enterococcus faecium SF68 dietary supplementation in overweight and obese cats without comorbidities. *Vet. Rec Open.* **3**, e000164. 10.1136/vetreco-2015-000164 (2016). 27110373 10.1136/vetreco-2015-000164 PMC4838762

[31] Ghosh, N., Wood, M. F. & Vitkin, I. A. Mueller matrix decomposition for extraction of individual polarization parameters from complex turbid media exhibiting multiple scattering, optical activity, and linear birefringence. *J. Biomed. Opt.* **13**, 044036. 10.1117/1.2960934 (2008). 19021363 10.1117/1.2960934

[32] Qiao, H. et al. Assessment of the physicochemical properties and bacterial composition of Lactobacillus plantarum and Enterococcus faecium-fermented Astragalus Membranaceus using single molecule, real-time sequencing technology. *Sci. Rep.* **8**, 11862. 10.1038/s41598-018-30288-x (2018). 30089930 10.1038/s41598-018-30288-x PMC6082834

[33] Salminen, M. K. et al. Lactobacillus bacteremia, clinical significance, and patient outcome, with special focus on probiotic L. rhamnosus GG. *Clin. Infect. Dis.* **38**, 62–69. 10.1086/380455 (2004). 14679449 10.1086/380455

[34] Barber, T. M., Kabisch, S., Pfeiffer, A. F. H. & Weickert, M. O. Dietary and lifestyle strategies for obesity. *Nutrients* **16**, 412. 10.3390/nu16162714 (2024). 10.3390/nu16162714 PMC11356871 39203850

[35] Ondee, T. et al. Lactiplantibacillus plantarum dfa1 outperforms Enterococcus faecium dfa1 on anti-obesity in high fat-induced obesity mice possibly through the differences in gut dysbiosis attenuation, despite the similar anti-Inflammatory properties. *Nutrients* **14**, 14523. 10.3390/nu14010080 (2021). 10.3390/nu14010080 PMC8746774 35010955

[36] Namjud, N. et al. Glycemic index and glycemic load of brief sugary sweets: randomized controlled trials of eight Thai desserts. *Front. Nutr.* **11**, 2024. 10.3389/fnut.2024.1452602 (2024). 10.3389/fnut.2024.1452602 PMC11557348 39539376

[37] Institute of Nutrition. *Institute of Nutrition, Mahidol University* (Nakhon Pathom, 2020).

[38] Sae-Khow, K. et al. Pathogen-Associated molecules from gut translocation enhance severity of cecal ligation and puncture Sepsis in Iron-Overload β-Thalassemia mice. *J. Inflamm. Res.* **13**, 719–735. 10.2147/jir.S273329 (2020). 33116751 10.2147/JIR.S273329 PMC7569041

[39] Thim-Uam, A. et al. Leaky-gut enhanced lupus progression in the Fc gamma receptor-IIb deficient and pristane-induced mouse models of lupus. *Sci. Rep.* **10**, 777. 10.1038/s41598-019-57275-0 (2020). 31964918 10.1038/s41598-019-57275-0 PMC6972921

[40] Visitchanakun, P. et al. Gut leakage enhances sepsis susceptibility in iron-overloaded β-thalassemia mice through macrophage hyperinflammatory responses. *Am. J. Physiol. Gastrointest. Liver Physiol.* **318**, G966–g979. 10.1152/ajpgi.00337.2019 (2020). 32308038 10.1152/ajpgi.00337.2019

[41] Panpetch, W. et al. Lactobacillus rhamnosus attenuates Thai Chili extracts induced gut inflammation and dysbiosis despite capsaicin bactericidal effect against the probiotics, a possible toxicity of high dose capsaicin. *PLoS One*. **16**, e0261189. 10.1371/journal.pone.0261189 (2021). 34941893 10.1371/journal.pone.0261189 PMC8699716

[42] Jin, L. et al. Impact of ferulic acid esterase-producing lactobacilli and fibrolytic enzymes on ensiling and digestion kinetics of mixed small-grain silage. *Grass Forage Sci.* **72**, 80–92. 10.1111/gfs.12217 (2017).

[43] Chancharoenthana, W. et al. Lacticaseibacilli attenuated fecal dysbiosis and metabolome changes in Candida-administered bilateral nephrectomy mice. *Front. Immunol.* **14**, 2023. 10.3389/fimmu.2023.1131447 (2023). 10.3389/fimmu.2023.1131447 PMC10034098 36969207

[44] Wang, G. et al. Lactobacillus rhamnosus strains relieve Loperamide-Induced constipation via different pathways independent of Short-Chain fatty acids. *Front. Cell. Infect. Microbiol.* **10**, 423. 10.3389/fcimb.2020.00423 (2020). 32974216 10.3389/fcimb.2020.00423 PMC7466723

[45] Savari, F., Mard, S. A., Badavi, M., Rezaie, A. & Gharib-Naseri, M. K. A new method to induce nonalcoholic steatohepatitis (NASH) in mice. *BMC Gastroenterol.* **19**, 125. 10.1186/s12876-019-1041-x (2019). 31307427 10.1186/s12876-019-1041-x PMC6632212

[46] Urai, T., Mukai, K., Matsushita, T. & Asano, K. The relationship between cutaneous wounds made on obese mice or those with decreased body weight and serum leptin level. *Health* **8**, 1015–1028. 10.4236/health.2016.811105 (2016).

[47] Klaikeaw, N., Wongphoom, J., Werawatganon, D., Chayanupatkul, M. & Siriviriyakul, P. Anti-inflammatory and anti-oxidant effects of aloe vera in rats with non-alcoholic steatohepatitis. *World J. Hepatol.* **12**, 363–377. 10.4254/wjh.v12.i7.363 (2020). 32821335 10.4254/wjh.v12.i7.363 PMC7407916

[48] Issara-Amphorn, J., Somboonna, N., Pisitkun, P., Hirankarn, N. & Leelahavanichkul, A. Syk inhibitor attenuates inflammation in lupus mice from FcgRIIb deficiency but not in pristane induction: the influence of lupus pathogenesis on the therapeutic effect. *Lupus* **29**, 1248–1262. 10.1177/0961203320941106 (2020). 32700597 10.1177/0961203320941106

[49] Schloss, P. D. et al. Introducing mothur: open-source, platform-independent, community-supported software for describing and comparing microbial communities. *Appl. Environ. Microbiol.* **75**, 7537–7541. 10.1128/aem.01541-09 (2009). 19801464 10.1128/AEM.01541-09 PMC2786419

[50] Bulan, D. E. et al. Spatial and seasonal variability of reef bacterial communities in the upper Gulf of Thailand. *Front. Mar. Sci.* **5**, 142. 10.3389/fmars.2018.00441 (2018).

[51] Segata, N. et al. Metagenomic biomarker discovery and explanation. *Genome Biol.* **12**, R60. 10.1186/gb-2011-12-6-r60 (2011). 21702898 10.1186/gb-2011-12-6-r60 PMC3218848

[52] Hiengrach, P., Panpetch, W., Chindamporn, A. & Leelahavanichkul, A. Helicobacter pylori, protected from antibiotics and stresses inside Candida albicans vacuoles, cause gastritis in mice. *Int. J. Mol. Sci.* **23**, 365. 10.3390/ijms23158568 (2022). 10.3390/ijms23158568 PMC9368807 35955701

[53] Sriwichaiin, S. et al. Deferiprone has less benefits on gut microbiota and metabolites in high iron-diet induced iron overload thalassemic mice than in iron overload wild-type mice: a preclinical study. *Life Sci.* **307**, 120871. 10.1016/j.lfs.2022.120871 (2022). 35952729 10.1016/j.lfs.2022.120871

[54] Wang, Z. Y. et al. Dextran sulfate sodium-induced gut microbiota dysbiosis aggravates liver injury in mice with S100-induced autoimmune hepatitis. *Immunol. Lett.* **263**, 70–77. 10.1016/j.imlet.2023.10.001 (2023). 37797724 10.1016/j.imlet.2023.10.001

[55] Zheng, Z. et al. Allobaculum involves in the modulation of intestinal ANGPTLT4 expression in mice treated by High-Fat diet. *Front. Nutr.* **8**, 690138. 10.3389/fnut.2021.690138 (2021). 34095196 10.3389/fnut.2021.690138 PMC8171929

[56] Xu, Y. et al. Function of Akkermansia muciniphila in obesity: interactions with lipid metabolism, immune response and gut systems. *Front. Microbiol.* **11**, 219. 10.3389/fmicb.2020.00219 (2020). 32153527 10.3389/fmicb.2020.00219 PMC7046546

[57] Savidge, T. C. et al. Lipopolysaccharide-induced human enterocyte tolerance to cytokine-mediated interleukin-8 production May occur independently of TLR-4/MD-2 signaling. *Pediatr. Res.* **59**, 89–95. 10.1203/01.pdr.0000195101.74184.e3 (2006). 16326999 10.1203/01.pdr.0000195101.74184.e3 PMC4465784

[58] Zhang, X. et al. Glucose but not Fructose alters the intestinal paracellular permeability in association with gut inflammation and dysbiosis in mice. *Front. Immunol.* **12**, 742584. 10.3389/fimmu.2021.742584 (2021). 35024040 10.3389/fimmu.2021.742584 PMC8744209

[59] Chen, L., Tuo, B. & Dong, H. Regulation of intestinal glucose absorption by ion channels and transporters. *Nutrients* **8**, 25. 10.3390/nu8010043 (2016). 10.3390/nu8010043 PMC4728656 26784222

[60] Havel, P. J. Dietary fructose: implications for dysregulation of energy homeostasis and lipid/carbohydrate metabolism. *Nutr. Rev.* **63**, 133–157. 10.1301/nr.2005.may.133-157 (2005). 15971409 10.1301/nr.2005.may.133-157

[61] Corpe, C. P., Burant, C. F. & Hoekstra, J. H. Intestinal Fructose absorption: clinical and molecular aspects. *J. Pediatr. Gastroenterol. Nutr.* **28**, 364–374. 10.1097/00005176-199904000-00004 (1999). 10204498 10.1097/00005176-199904000-00004

[62] Jang, C. et al. The small intestine converts dietary Fructose into glucose and organic acids. *Cell. Metab.* **27**, 351–361e353. 10.1016/j.cmet.2017.12.016 (2018). 29414685 10.1016/j.cmet.2017.12.016 PMC6032988

[63] Cawley, N. X. Sugar making sugar: gluconeogenesis triggered by Fructose via a hypothalamic-adrenal-corticosterone circuit. *Endocrinology* **153**, 3561–3563. 10.1210/en.2012-1562 (2012). 22822224 10.1210/en.2012-1562 PMC3404346

[64] Andres-Hernando, A., Johnson, R. J. & Lanaspa, M. A. Endogenous Fructose production: what do we know and how relevant is it? *Curr. Opin. Clin. Nutr. Metab. Care*. **22**, 289–294. 10.1097/mco.0000000000000573 (2019). 31166222 10.1097/MCO.0000000000000573 PMC6684314

[65] Lanaspa, M. A. et al. Endogenous Fructose production and metabolism in the liver contributes to the development of metabolic syndrome. *Nat. Commun.* **4**, 2434. 10.1038/ncomms3434 (2013). 24022321 10.1038/ncomms3434 PMC3833672

[66] Perez-Pozo, S. E. et al. Excessive Fructose intake induces the features of metabolic syndrome in healthy adult men: role of uric acid in the hypertensive response. *Int. J. Obes. (Lond)*. **34**, 454–461. 10.1038/ijo.2009.259 (2010). 20029377 10.1038/ijo.2009.259

[67] Jegatheesan, P., De Bandt, J. P. & Fructose The multifaceted aspects of Fructose metabolism. *Nutrients* **9**, 47. 10.3390/nu9030230 (2017).

[68] Haukeland, J. W. et al. Abnormal glucose tolerance is a predictor of steatohepatitis and fibrosis in patients with non-alcoholic fatty liver disease. *Scand. J. Gastroenterol.* **40**, 1469–1477. 10.1080/00365520500264953 (2005). 16293559 10.1080/00365520500264953

[69] Seo, Y. S., Lee, H. B., Kim, Y. & Park, H. Y. Dietary carbohydrate constituents related to gut dysbiosis and health. *Microorganisms* **8**, 745. 10.3390/microorganisms8030427 (2020). 10.3390/microorganisms8030427 PMC7143995 32197401

[70] González-Garcinuño, Á., Tabernero, A., Sánchez-Álvarez, J. M. & Galán, M. A. Effect of bacteria type and sucrose concentration on Levan yield and its molecular weight. *Microb. Cell. Fact.* **16**, 91. 10.1186/s12934-017-0703-z (2017). 28535808 10.1186/s12934-017-0703-z PMC5442672

[71] Balay, D. R., Gänzle, M. G. & McMullen, L. M. The effect of carbohydrates and bacteriocins on the growth kinetics and resistance of Listeria monocytogenes. *Front. Microbiol.* **9**, 347. 10.3389/fmicb.2018.00347 (2018). 29545781 10.3389/fmicb.2018.00347 PMC5838005

[72] Huang, J., Gao, K., Yang, L. & Lu, Y. Successional action of bacteroidota and Firmicutes in decomposing straw polymers in a paddy soil. *Environ. Microbiome*. **18**, 76. 10.1186/s40793-023-00533-6 (2023). 37838745 10.1186/s40793-023-00533-6 PMC10576277

[73] Froidurot, A. & Julliand, V. Cellulolytic bacteria in the large intestine of mammals. *Gut Microbes*. **14**, 2031694. 10.1080/19490976.2022.2031694 (2022). 35184689 10.1080/19490976.2022.2031694 PMC8865330

[74] Chen, H. et al. Lactobacillus plantarum HF02 alleviates lipid accumulation and intestinal microbiota dysbiosis in high-fat diet-induced obese mice. *J. Sci. Food Agric.* **103**, 4625–4637. 10.1002/jsfa.12538 (2023). 36866521 10.1002/jsfa.12538

[75] Kobyliak, N. et al. Probiotics in prevention and treatment of obesity: a critical view. *Nutr. Metab. (Lond)*. **13**, 14. 10.1186/s12986-016-0067-0 (2016). 26900391 10.1186/s12986-016-0067-0 PMC4761174

[76] Chen, J., Chen, X. & Ho, C. L. Recent development of probiotic bifidobacteria for treating human diseases. *Front. Bioeng. Biotechnol.* **9**, 770248. 10.3389/fbioe.2021.770248 (2021). 35004640 10.3389/fbioe.2021.770248 PMC8727868

[77] Kelly, S. M. & Munoz-Munoz, J. Sinderen, D. Plant glycan metabolism by bifidobacteria. *Front. Microbiol.* **12**, 609418. 10.3389/fmicb.2021.609418 (2021). van. 33613480 10.3389/fmicb.2021.609418 PMC7889515

[78] Salles, B. I. M., Cioffi, D. & Ferreira, S. R. G. Probiotics supplementation and insulin resistance: a systematic review. *Diabetol. Metab. Syndr.* **12**, 98. 10.1186/s13098-020-00603-6 (2020). 33292434 10.1186/s13098-020-00603-6 PMC7656736

[79] Pintarič, M. & Langerholc, T. Probiotic mechanisms affecting glucose homeostasis: A scoping review. *Life (Basel)*. **12**, 1423. 10.3390/life12081187 (2022). 10.3390/life12081187 PMC9409775 36013366

[80] Liu, M. J. et al. Recent findings in Akkermansia muciniphila-regulated metabolism and its role in intestinal diseases. *Clin. Nutr.* **41**, 2333–2344. 10.1016/j.clnu.2022.08.029 (2022). 36113229 10.1016/j.clnu.2022.08.029

[81] Engevik, M. A. et al. Bifidobacterium dentium Fortifies the Intestinal Mucus Layer via Autophagy and Calcium Signaling Pathways. *mBio* **10**, 412. 10.1128/mBio.01087-19 (2019). 10.1128/mBio.01087-19 PMC6581858 31213556

[82] Holmberg, S. M. et al. The gut commensal Blautia maintains colonic mucus function under low-fiber consumption through secretion of short-chain fatty acids. *Nat. Commun.* **15**, 3502. 10.1038/s41467-024-47594-w (2024). 38664378 10.1038/s41467-024-47594-w PMC11045866

[83] Thaiss, C. A. et al. Hyperglycemia drives intestinal barrier dysfunction and risk for enteric infection. *Science* **359**, 1376–1383. 10.1126/science.aar3318 (2018). 29519916 10.1126/science.aar3318

[84] Matuschik, L. et al. Hyperglycemia induces inflammatory response of human macrophages to CD163-Mediated scavenging of Hemoglobin-Haptoglobin complexes. *Int. J. Mol. Sci.* **23**, 475. 10.3390/ijms23031385 (2022). 10.3390/ijms23031385 PMC8836198 35163309

[85] Charoensappakit, A., Sae-Khow, K. & Leelahavanichkul, A. Gut barrier damage and gut translocation of pathogen molecules in lupus, an impact of innate immunity (Macrophages and Neutrophils) in autoimmune disease. *Int. J. Mol. Sci.* **23**, 96. 10.3390/ijms23158223 (2022). 10.3390/ijms23158223 PMC9367802 35897790

