# Supplementary material for: Lactiplantibacillus plantarum dfa1 reduces obesity caused by a high carbohydrate diet by modulating inflammation and gut microbiota
Source: Sci Rep. 2025 Jul 10;15:24801. doi: 10.1038/s41598-025-10435-x (PMC12241623; doi:10.1038/s41598-025-10435-x)
Supplement: Supplementary file 1 — Supplementary Material 1 [file 41598_2025_10435_MOESM1_ESM.docx]

**Supplementary Appendix: Detailed Composition of Experimental Diets and Nutritional Information.**

**Table A1** Nutrient composition per 15 kcal/day of dietary interventions (reflecting the average daily intake in mice).

| **Dietary interventions** | **Energy (kcal/day)** | **Carbohydrate (g)** | **Protein (g)** | **Fat (g)** | **Crude Fiber (g)** | **Dietary Fiber (g)** |
| --- | --- | --- | --- | --- | --- | --- |
| Control diet | 15 | 2.08 | 1.17 | 0.22 | 0.24 | - |
| High Glucose Diet (HGD) | 15 | 2.49 | 0.89 | 0.17 | 0.18 | - |
| High-carbohydrate Biscuit Diet (HBD) | 15 | 2.63 | 0.32 | 0.35 | 0.04 | 0.68 |

**Table A2** Nutrient composition per 100 g of dietary interventions (standardized for comparison).

| **Dietary interventions** | **Energy (kcal)** | **Carbohydrate (g)** | **Protein (g)** | **Fat (g)** | **Crude Fiber (g)** | **Dietary Fiber (g)** |
| --- | --- | --- | --- | --- | --- | --- |
| Control diet | 306.70 | 42.55 | 24.00 | 4.50 | 5.00 | - |
| High Glucose Diet (HGD) | 324.96 | 53.94 | 19.20 | 3.60 | 4.00 | - |
| High-carbohydrate Biscuit Diet (HBD) | 388.02 | 68.11 | 8.38 | 9.12 | 1.00 | 17.62 |

**Table A3** Ingredient proportions by weight (%) in each dietary intervention.

|  | **Control Diet (%)** | **High Glucose Diet (%)** | **High-carbohydrate Biscuit Diet (%)** |
| --- | --- | --- | --- |
| Standard Laboratory Chow (CP#082) | 100 | 80 | 20 |
| Glucose (powder) | - | 20 | - |
| Pineapple Cheese Cake Biscuit | - | - | 80 |

Composition of Pineapple Cheese Cake Biscuit (Suphalak Brand**:** Pineapple jam (50%), Wheat flour (45%), Sugar (4%), and Salt (1%).

**Note:**

- Nutrient value of Pineapple Cheese Cake Biscuit were obtained from: *Namjud N, Senaprom S, Ondee T, Bumrungpert A, Heath J, Pongpirul K. Glycemic index and glycemic load of brief sugary sweets: randomized controlled trials of eight Thai desserts. Frontiers in Nutrition, 2024.*
- Dietary fiber content was derived from INMUCAL-nutrients V.4.0, *Institute of Nutrition, Mahidol University, 2020.*
- Crude fiber and macronutrient values for the standard laboratory chow were sourced from the manufacturer’s product label (CP#082).
