# Supplementary figures and images for: Lactiplantibacillus plantarum dfa1 reduces obesity caused by a high carbohydrate diet by modulating inflammation and gut microbiota
Source: Sci Rep. 2025 Jul 10;15:24801. doi: 10.1038/s41598-025-10435-x (PMC12241623; doi:10.1038/s41598-025-10435-x)

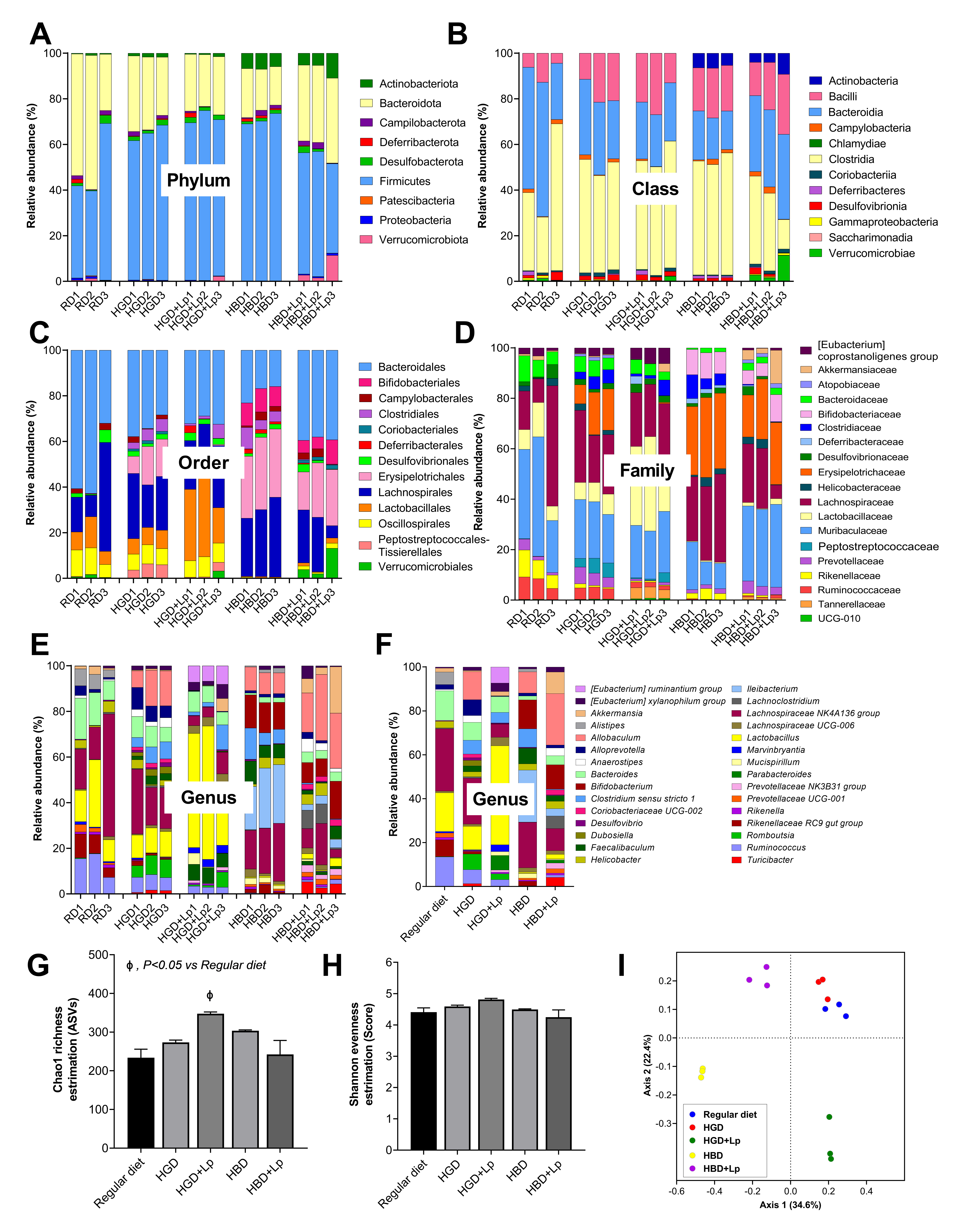

Supplement: Supplementary file 2 — Supplementary Material 2 [file 41598_2025_10435_MOESM2_ESM.tif]

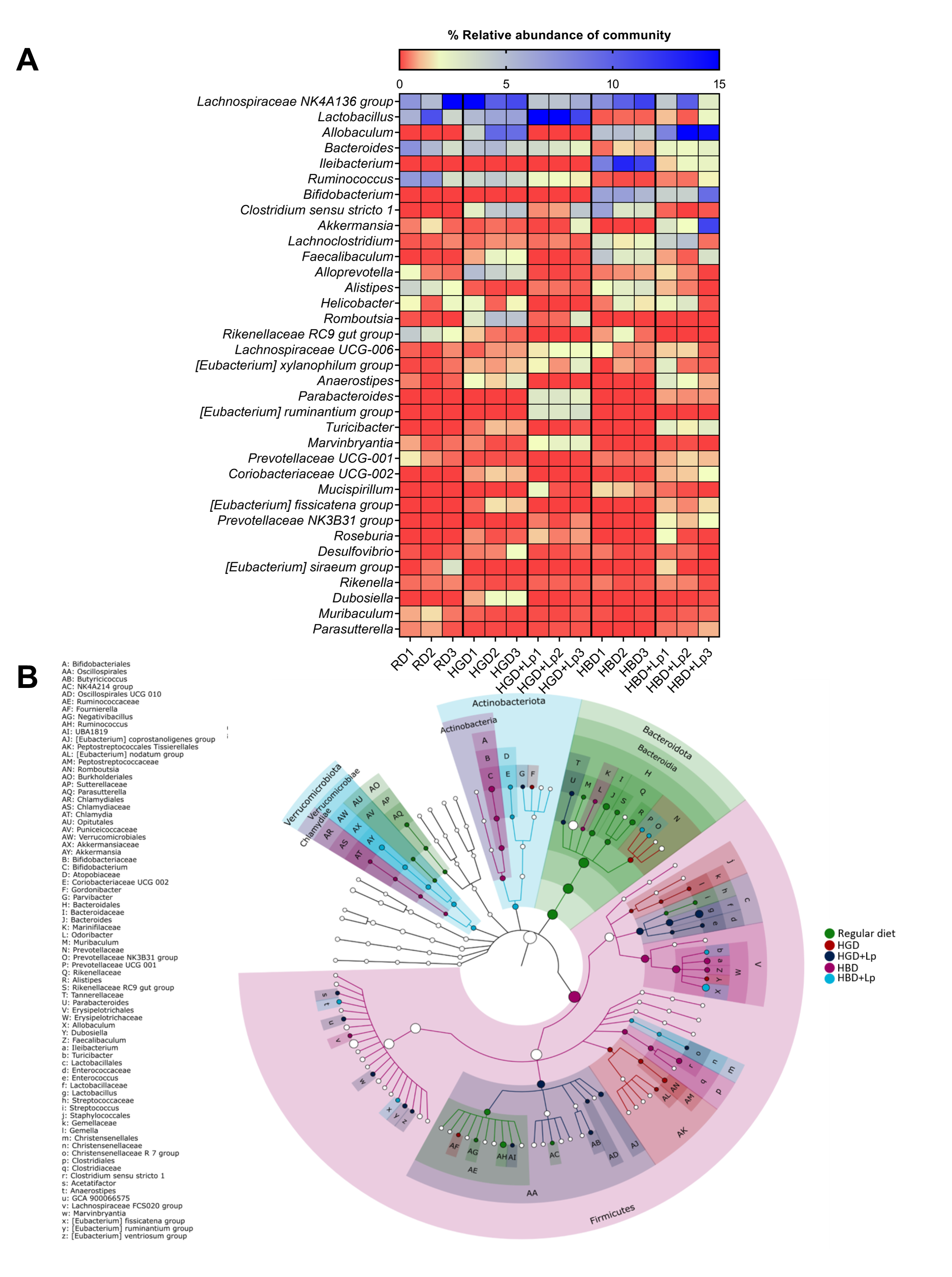

Supplement: Supplementary file 3 — Supplementary Material 3 [file 41598_2025_10435_MOESM3_ESM.tif]
