# Supplementary material for: Lactiplantibacillus plantarum dfa1 reduces obesity caused by a high carbohydrate diet by modulating inflammation and gut microbiota
Source: Sci Rep. 2025 Jul 10;15:24801. doi: 10.1038/s41598-025-10435-x (PMC12241623; doi:10.1038/s41598-025-10435-x)
Supplement: Supplementary file 4 — Supplementary Material 4 [file 41598_2025_10435_MOESM4_ESM.docx]

**Supplement Figure 1.** Taxonomic changes in gut microbiota composition in response to regular diet (RD), high glucose diet (HGD), or high-carbohydrate biscuit diet (HBD), with or without *Lactiplantibacillus plantarum* (Lp) dfa1: The details of relative abundance of bacteria in different level comparison, including phylum, class, order, family, genus, and the average values of the abundance in genus level) (A-F), alpha diversity assessed by Chao1 richness and Shannon evenness indices (G, H). and the non-metric multidimensional scaling (NMDS) plot for beta diversity (I) are demonstrated.

**Supplement Figure 2.** Taxonomic changes in gut microbiota composition in response to regular diet (RD), high glucose diet (HGD), or high-carbohydrate biscuit diet (HBD), with or without *Lactiplantibacillus plantarum* (Lp) dfa1: Heatmap showing genus-level abundance (A) and Cladogram (the diagram demonstrating the evolutionary relationships among different groups of organisms) (B) are demonstrated.
