# Supplementary material for: Lactiplantibacillus plantarum dfa1 reduces obesity caused by a high carbohydrate diet by modulating inflammation and gut microbiota
Source: Sci Rep. 2025 Jul 10;15:24801. doi: 10.1038/s41598-025-10435-x (PMC12241623; doi:10.1038/s41598-025-10435-x)
Supplement: Supplementary file 5 — Supplementary Material 5 [file 41598_2025_10435_MOESM5_ESM.docx]

**Table 1** Comparison of fecal microbiota in genus level between regular diet vs high glucose diet (HGD) mice.

| **Bacteria** | **Relative abundance (%)** | | **p-value** | **Direction*** |
| --- | --- | --- | --- | --- |
|  | **Regular diet** | **HGD** |  |  |
| *[Eubacterium] fissicatena group* | 0.000 | 1.290 | 0.039 | Up |
| *Alistipes* | 6.591 | 1.132 | 0.043 | Down |
| *Allobaculum* | 0.000 | 11.725 | 0.013 | Up |
| *Anaerostipes* | 0.472 | 2.537 | 0.022 | Up |
| *Christensenellaceae R-7 group* | 0.000 | 0.132 | 0.002 | Up |
| *Clostridium sensu stricto 1* | 0.000 | 5.547 | 0.005 | Up |
| *Coriobacteriaceae UCG-002* | 0.000 | 1.376 | 0.001 | Up |
| *Dubosiella* | 0.000 | 2.081 | 0.007 | Up |
| *Faecalibaculum* | 0.054 | 2.279 | 0.016 | Up |
| *Gemella* | 0.000 | 0.041 | 0.002 | Up |
| *Gordonibacter* | 0.039 | 0.189 | 0.019 | Up |
| *Lachnospiraceae UCG-001* | 0.039 | 0.349 | 0.002 | Up |
| *Monoglobus* | 0.078 | 0.008 | 0.010 | Down |
| *Negativibacillus* | 0.157 | 0.047 | 0.005 | Down |
| *Rikenella* | 0.808 | 0.203 | 0.003 | Down |
| *Romboutsia* | 0.135 | 5.918 | 0.004 | Up |
| *Turicibacter* | 0.000 | 1.086 | 0.017 | Up |

*Direction of change in HGD compared with regular diet

**Table 2** Comparison of fecal microbiota in genus level between regular diet vs high-carbohydrate biscuit diet (HBD) mice.

| **Bacteria** | **Relative abundance (%)** | | **p-value** | **Direction*** |
| --- | --- | --- | --- | --- |
|  | **Regular diet** | **HBD** |  |  |
| *[Eubacterium] nodatum group* | 0.196 | 0.051 | 0.002 | Down |
| *Acetatifactor* | 0.091 | 0.333 | 0.010 | Up |
| *Allobaculum* | 0.000 | 7.991 | 0.0001 | Up |
| *Anaeroplasma* | 0.000 | 0.077 | 0.021 | Up |
| *Bacteroides* | 10.454 | 1.555 | 0.021 | Down |
| *Bifidobacterium* | 0.000 | 9.537 | 0.046 | Up |
| *Bilophila* | 0.000 | 0.048 | <0.001 | Up |
| *Chlamydia* | 0.000 | 0.045 | 0.017 | Up |
| *Clostridium sensu stricto 1* | 0.000 | 6.456 | 0.034 | Up |
| *Faecalibaculum* | 0.054 | 5.592 | 0.011 | Up |
| *GCA-900066575* | 0.267 | 1.307 | 0.01 | Up |
| *Ileibacterium* | 0.000 | 17.076 | 0.001 | Up |
| *Intestinimonas* | 0.101 | 0.511 | 0.005 | Up |
| *Lachnospiraceae FCS020 group* | 0.077 | 0.835 | 0.001 | Up |
| *Monoglobus* | 0.078 | 0.012 | 0.007 | Down |
| *Negativibacillus* | 0.157 | 0.100 | 0.009 | Down |
| *Odoribacter* | 0.000 | 0.801 | 0.001 | Up |
| *Ruminococcus* | 0.542 | 6.506 | 0.020 | Up |

*Direction of change in HBD compared with regular diet

**Table 3** Comparison of fecal microbiota in genus level between high glucose diet (HGD) vs probiotics-treated HGD (HGD+Lp) mice.

| **Bacteria** | **Relative abundance (%)** | | **p-value** | **Direction*** |
| --- | --- | --- | --- | --- |
|  | **HGD** | **HGD+Lp** |  |  |
| *[Eubacterium] fissicatena group* | 1.290 | 0.000 | 0.039 | Down |
| *[Eubacterium] ruminantium group* | 0.000 | 4.814 | <0.001 | Up |
| *Allobaculum* | 11.725 | 0.000 | 0.013 | Down |
| *Alloprevotella* | 5.917 | 0.132 | 0.004 | Down |
| *Anaerostipes* | 2.537 | 0.000 | 0.008 | Down |
| *Anaerotruncus* | 0.213 | 0.819 | <0.001 | Up |
| *ASF356* | 0.505 | 1.196 | 0.029 | Up |
| *Bilophila* | 0.000 | 0.057 | 0.028 | Up |
| *Butyricicoccus* | 0.358 | 1.446 | <0.001 | Up |
| *Coriobacteriaceae UCG-002* | 1.376 | 0.000 | 0.001 | Down |
| *Dubosiella* | 2.081 | 0.058 | 0.007 | Down |
| *Erysipelatoclostridium* | 0.091 | 0.508 | 0.040 | Up |
| *Faecalibaculum* | 2.279 | 0.058 | 0.016 | Down |
| *Incertae Sedis* | 0.239 | 0.772 | 0.006 | Up |
| *Intestinimonas* | 0.126 | 0.482 | 0.037 | Up |
| *Lachnospiraceae UCG-006* | 0.853 | 2.629 | 0.002 | Up |
| *Lactobacillus* | 8.814 | 31.620 | 0.018 | Up |
| *Marvinbryantia* | 0.408 | 3.232 | 0.005 | Up |
| *Odoribacter* | 0.000 | 0.192 | 0.005 | Up |
| *Parabacteroides* | 0.000 | 4.352 | 0.021 | Up |
| *Parasutterella* | 0.494 | 0.214 | 0.027 | Down |
| *Prevotellaceae UCG-001* | 0.732 | 0.033 | 0.001 | Down |
| *Rikenella* | 0.203 | 0.414 | 0.012 | Up |
| *Ruminococcus* | 5.713 | 2.693 | 0.002 | Down |
| *Streptococcus* | 0.008 | 0.113 | 0.023 | Up |
| *Turicibacter* | 1.086 | 0.081 | 0.025 | Down |
| *Tuzzerella* | 0.105 | 0.375 | 0.009 | Up |
| *UCG-009* | 0.108 | 0.470 | 0.029 | Up |

*Direction of change in HGD+Lp compared with HGD

**Table 4** Comparison of fecal microbiota in genus level between high-carbohydrate biscuit diet (HBD) vs probiotics-treated HBD (HBD+Lp) mice**.**

| **Bacteria** | **Relative abundance (%)** | | **p-value** | **Direction*** |
| --- | --- | --- | --- | --- |
|  | **HBD** | **HBD+Lp** |  |  |
| *[Eubacterium] fissicatena group* | 0.000 | 1.450 | 0.010 | Up |
| *Acetatifactor* | 0.333 | 0.015 | <0.001 | Down |
| *Akkermansia* | 0.000 | 8.245 | 0.007 | Up |
| *Alistipes* | 3.879 | 1.362 | 0.032 | Down |
| *Allobaculum* | 7.991 | 21.576 | 0.020 | Up |
| *Anaerostipes* | 0.000 | 2.720 | 0.030 | Up |
| *Anaerovorax* | 0.043 | 0.005 | 0.005 | Down |
| *Bacteroides* | 1.555 | 3.489 | 0.014 | Up |
| *Chlamydia* | 0.045 | 0.000 | 0.017 | Down |
| *Clostridium sensu stricto 1* | 6.456 | 0.240 | 0.038 | Down |
| *Coriobacteriaceae UCG-002* | 0.025 | 1.878 | 0.002 | Up |
| *Enterorhabdus* | 0.440 | 0.169 | 0.015 | Down |
| *Family XIII AD3011 group* | 0.000 | 0.084 | 0.050 | Up |
| *GCA-900066575* | 1.307 | 0.419 | 0.032 | Down |
| *Ileibacterium* | 17.076 | 2.701 | 0.001 | Down |
| *Lachnospiraceae FCS020 group* | 0.835 | 0.273 | 0.024 | Down |
| *Mucispirillum* | 1.533 | 0.263 | 0.021 | Down |
| *Negativibacillus* | 0.100 | 0.015 | 0.011 | Down |
| *Oscillibacter* | 0.913 | 0.391 | 0.017 | Down |
| *Parasutterella* | 0.170 | 0.932 | 0.033 | Up |
| *Prevotellaceae NK3B31 group* | 0.000 | 2.094 | 0.001 | Up |
| *Prevotellaceae UCG-001* | 0.626 | 1.584 | 0.003 | Up |
| *Turicibacter* | 0.002 | 3.304 | 0.005 | Up |

*Direction of change in HBD+Lp compared with HBD

**Table 5** Comparison of fecal microbiota in genus level between regular diet vs probiotics-treated HGD (HGD+Lp) mice.

| **Bacteria** | **Relative abundance (%)** | | **p-value** | **Direction*** |
| --- | --- | --- | --- | --- |
|  | **Regular diet** | **HGD+Lp** |  |  |
| *[Eubacterium] ruminantium group* | 0.000 | 4.814 | <0.001 | Up |
| *[Eubacterium] ventriosum group* | 0.000 | 0.096 | 0.031 | Up |
| *Alistipes* | 6.591 | 1.283 | 0.046 | Down |
| *Anaerotruncus* | 0.285 | 0.819 | 0.045 | Up |
| *Bilophila* | 0.000 | 0.057 | 0.028 | Up |
| *Butyricicoccus* | 0.408 | 1.446 | 0.005 | Up |
| *Fournierella* | 0.000 | 0.089 | 0.025 | Up |
| *GCA-900066575* | 0.267 | 1.325 | 0.013 | Up |
| *Incertae Sedis* | 0.175 | 0.772 | 0.008 | Up |
| *Intestinimonas* | 0.101 | 0.482 | 0.039 | Up |
| *Lachnospiraceae FCS020 group* | 0.077 | 0.420 | 0.005 | Up |
| *Lachnospiraceae UCG-006* | 0.836 | 2.629 | 0.017 | Up |
| *Marvinbryantia* | 0.811 | 3.232 | 0.012 | Up |
| *Odoribacter* | 0.000 | 0.192 | 0.005 | Up |
| *Parabacteroides* | 0.000 | 4.352 | 0.021 | Up |
| *Rikenella* | 0.808 | 0.414 | 0.011 | Down |
| *Rikenellaceae RC9 gut group* | 5.603 | 0.000 | 0.019 | Down |
| *Ruminococcus* | 10.944 | 2.693 | 0.045 | Down |

*Direction of change in HGD+Lp compared with regular diet

**Table 6** Comparison of fecal microbiota in genus level between regular diet vs probiotics-treated HBD (HBD+Lp) mice.

| **Bacteria** | **Relative abundance (%)** | | **p-value** | **Direction*** |
| --- | --- | --- | --- | --- |
|  | **Regular diet** | **HBD+Lp** |  |  |
| *[Eubacterium] fissicatena group* | 0.000 | 1.450 | 0.010 | Up |
| *[Eubacterium] nodatum group* | 0.196 | 0.107 | 0.039 | Down |
| *Allobaculum* | 0.000 | 21.576 | 0.004 | Up |
| *Bacteroides* | 10.454 | 3.489 | 0.045 | Down |
| *Bifidobacterium* | 0.000 | 8.999 | 0.028 | Up |
| *Coriobacteriaceae UCG-002* | 0.000 | 1.878 | 0.002 | Up |
| *Ileibacterium* | 0.000 | 2.701 | 0.003 | Up |
| *Negativibacillus* | 0.157 | 0.015 | 0.001 | Down |
| *Prevotellaceae NK3B31 group* | 0.000 | 2.094 | 0.001 | Up |
| *Rikenellaceae RC9 gut group* | 5.603 | 0.036 | 0.020 | Down |
| *Ruminococcus* | 10.944 | 1.207 | 0.028 | Down |
| *Turicibacter* | 0.000 | 3.304 | 0.005 | Up |

*Direction of change in HBD+Lp compared with regular diet
